# Supplementary material for: Sorghum embryos undergoing B chromosome elimination express B-variants of mitotic-related genes
Source: Genome Biol. 2025 Dec 24;27:8. doi: 10.1186/s13059-025-03915-w (PMC12849586; doi:10.1186/s13059-025-03915-w)
Supplement: Supplementary file 1 — Additional file 1. Supplementary Document: Figs. S1-S7, Tables S1-S3, (pipeline of transcriptomics experiments, LCM of sections dataset, additional PCA clustering and GSEA analysisof sections dataset, electrophoretogram of the B-specific amplicons, expression levels of B-localized genes, sequence content in S. purpureosericeum, primers for B-specific repeat clusters and candidates screening). [file 13059_2025_3915_MOESM1_ESM.pdf]

Sorghum embryos undergoing B chromosome elimination express B-variants of mitotic-related genes

Tereza Bojdová[1,2] (0009-0004-7257-270X), Lucie Hloušková[1,2] (0000-0003-4800-8186), Kateřina Holušová[1], Radim Svačina[1] (0000-0002-6481-4076), Eva Hřibová[1] (0000-0002-6868-4344), Iva Ilíková[1] (0000-0002-3547-3110), Johannes Thiel[3] (0000-0003-3924-6940), Gihwan Kim[3] (0000-0002-4621-1570), Roman Pleskot[4] (0000-0003-0436-9748), Andreas Houben[3] (0000-0003-3419-239X), Jan Bartoš[1]* (0000-0002-4154-8895) and Miroslava Karafiátová[1]* (0000-0003-1177-6472)

[1] Institute of Experimental Botany of the Czech Academy of Sciences, Centre of Plant Structural and Functional Genomics, Šlechtitelů 31, 779 00 Olomouc, Czech Republic
[2] Department of Cell Biology and Genetics, Faculty of Science, Palacky University, Šlechtitelů 27, 779 00 Olomouc, Czech Republic
[3] Leibniz Institute of Plant Genetics and Crop Plant Research (IPK), 06466 Seeland OT Gatersleben, Germany
[4] Institute of Experimental Botany of the Czech Academy of Sciences, Laboratory of Integrative Structural Biology, Rozvojová 263,165 00 Praha 6 - Lysolaje, Czech Republic

*Correspondence: bartos@ueb.cas.cz; karafiatova@ueb.cas.cz

Additional file 1: Supplementary Document: Fig. S1-S7, Table S1-S3 (file contains separate channels of FISH experiments (Fig. S1), pipeline of transcriptomics experiments (Fig. S2), LCM of sections dataset (Fig. S3), additional PCA clustering (Fig. S4) and GSEA analysis (Fig. S5) of sections dataset, electrophoretogram of the B -specific amplicons (Fig. S6), expression levels of B-localized genes (Fig. S7), sequence content in *S. purpureosericeum* (Table S1), primers for B-specific repeat clusters (Table S2) and candidates screening (Table S3)).

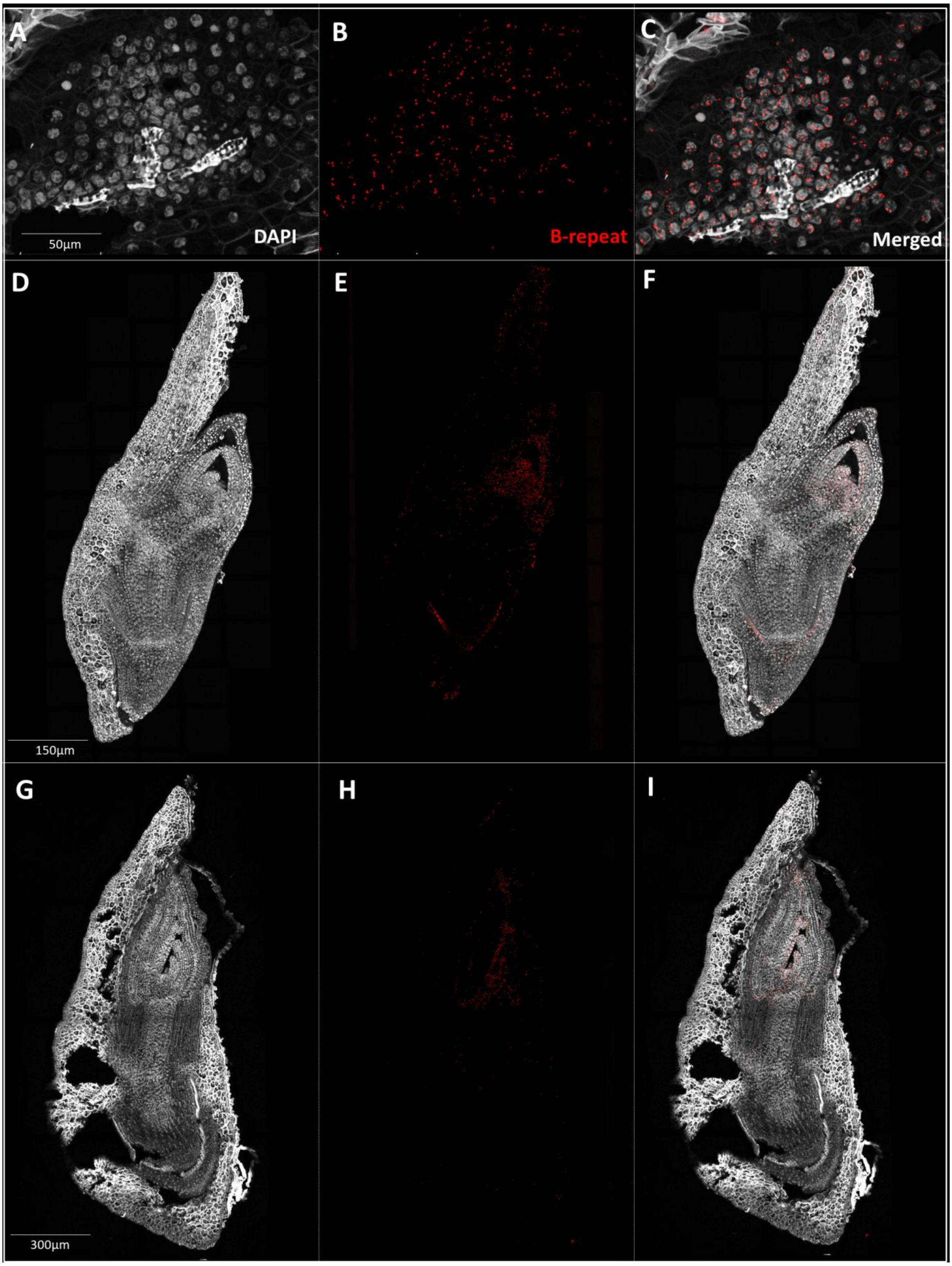

Fig. S1: In situ visualization of the B chromosome on embryo cryo-section during studied stages of embryonal development in wild sorghum. A-C – early stage; D-F – mid-stage; G-I – late stage. B-positive cells were detected using B-specific probe (red). DNA is counterstained with DAPI (grey*).* High resolution images have been submitted to the Zenodo repository (10.5281/zenodo.17812202).

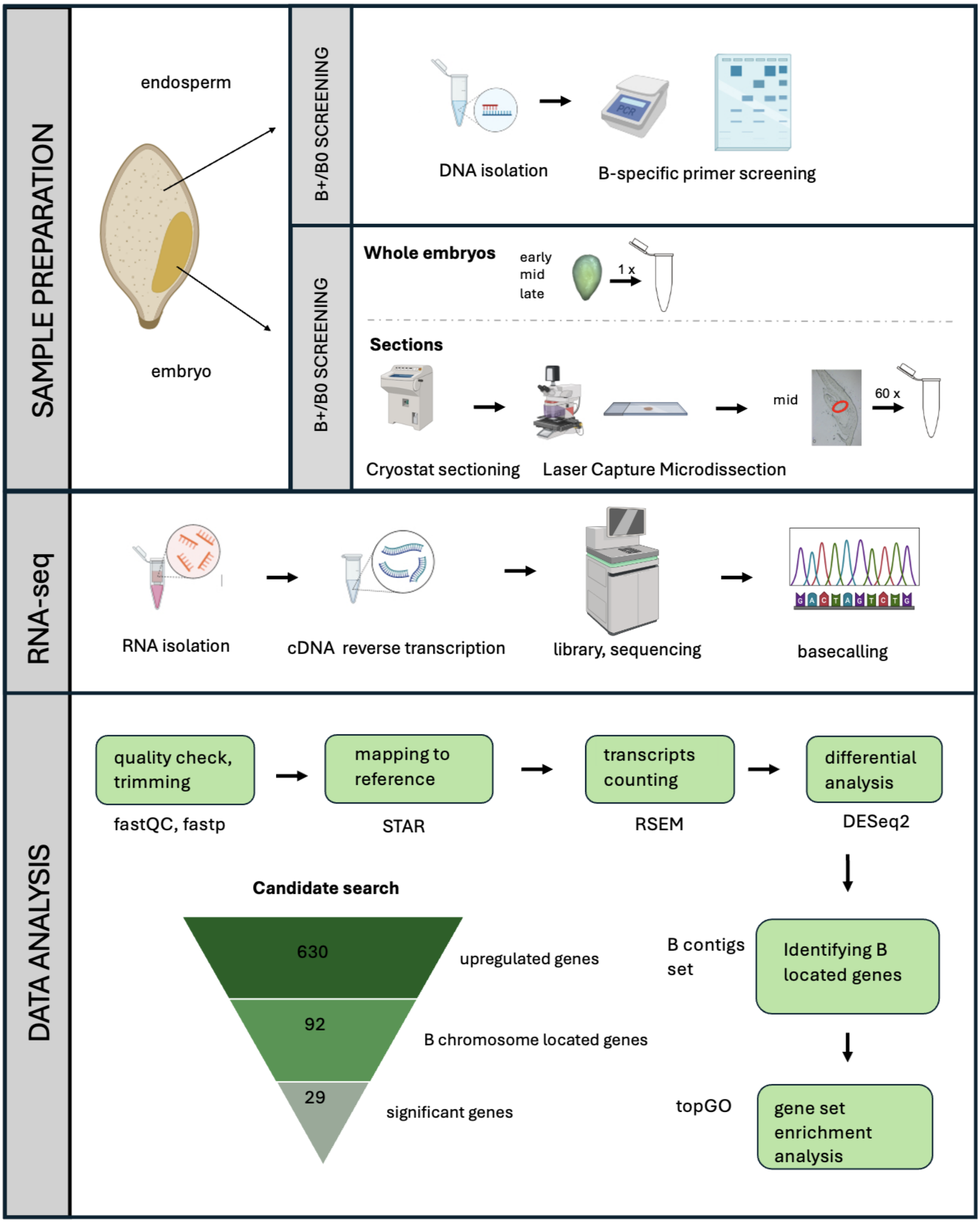

Fig. S2: Pipeline of transcriptomic experiments. Created with BioRender.com

A B

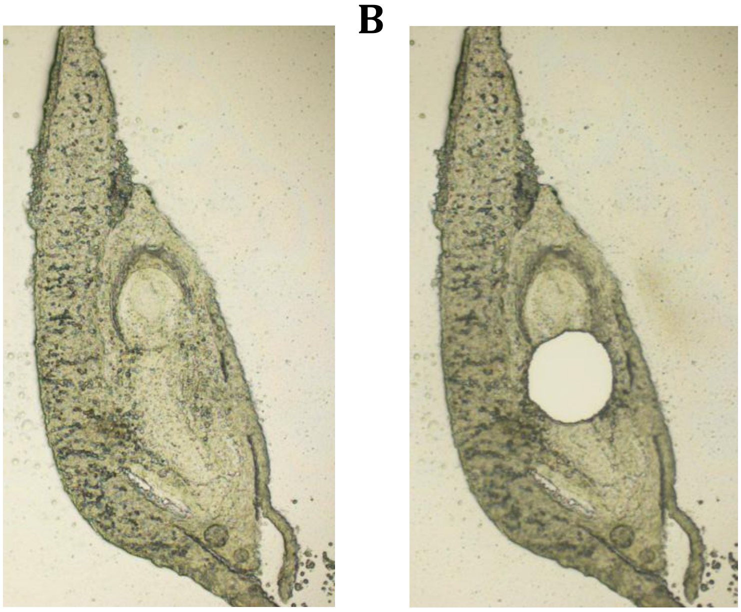

Fig. S3: Laser Capture Microdissection of embryonic region undergoing B chromosome elimination. A – Mid-stage embryo section before dissection, B – Mid-stage embryo section after dissection.

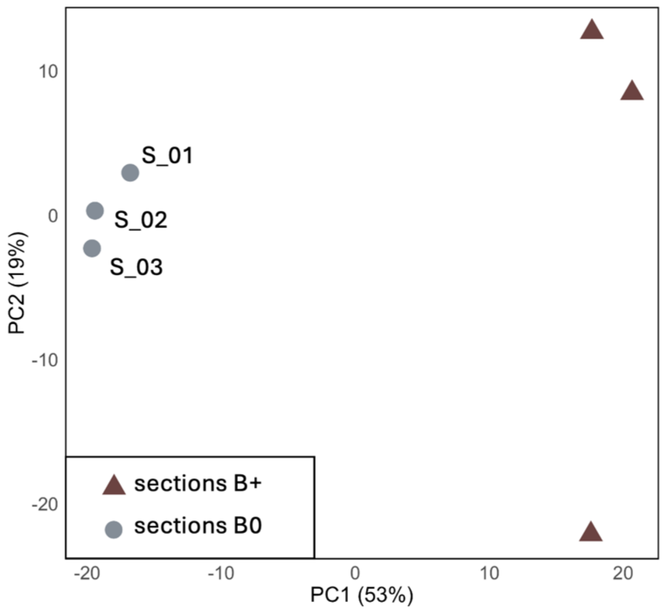

Fig. S4: Principal Component Analysis (PCA) variance of 6 section RNA-seq samples.

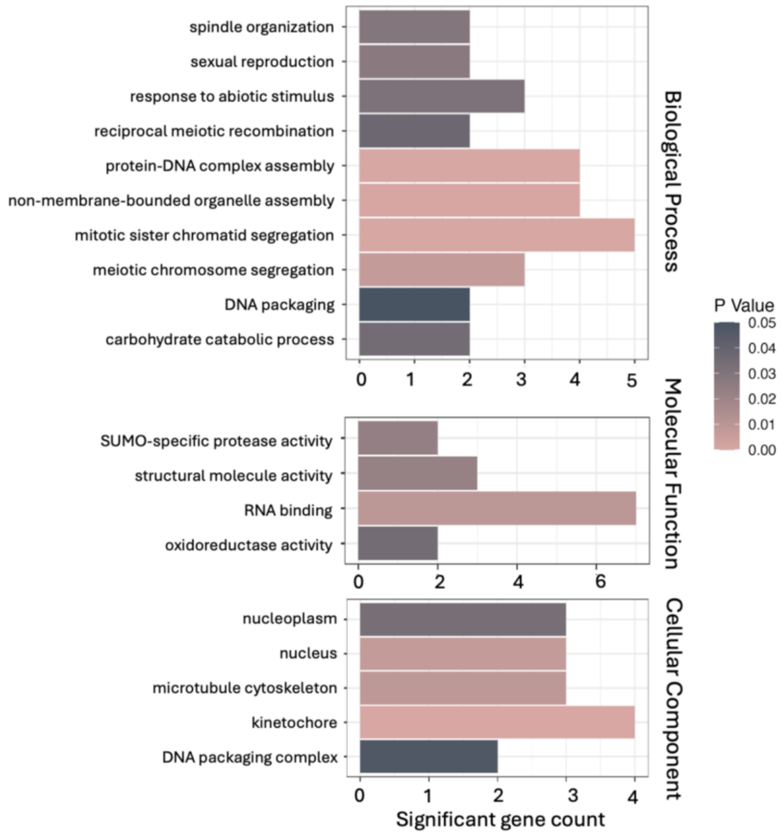

Fig. S5: Gene set enrichment analysis of upregulated B located genes in the sections dataset. The barplot visualizes the enrichment of all significantly enriched gene ontology (GO) terms (p-value < 0.05). Bar length represent number of identified upregulated genes; colour correspond to significance level.

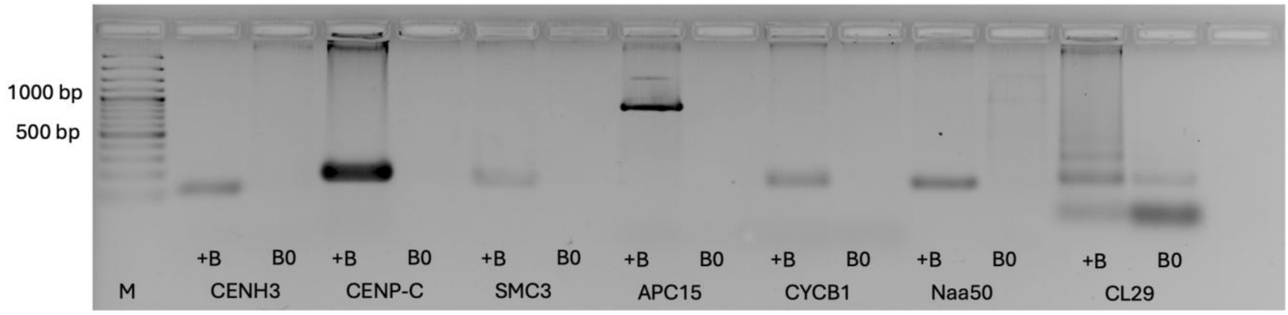

Fig. S6: B-specific PCR amplicons for candidates using B+ and B0 DNA templates. Due to impossibility to design B-specific primer for gene sequence, downstream sequence of utg10093 is included. GeneRuler 100 bp DNA Ladder was used for size reference. As a negative control, centromeric repetition CL29 (Karafiátová et al., 2024) was used.

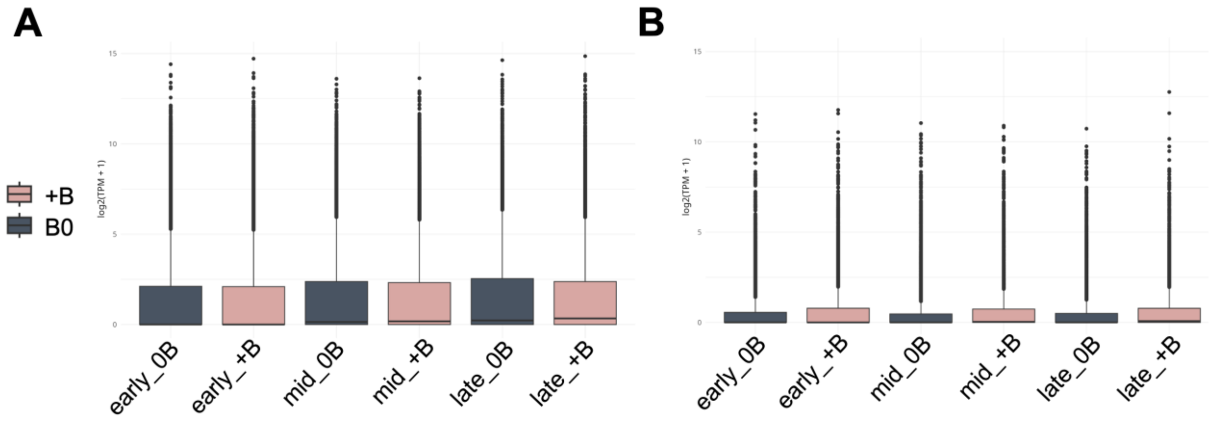

Fig. S7: Boxplot showing the log (TPM+1) transformed expression levels of all genes (**A**) and B-localized genes (**B**).

Table S1: Sequence content in *S. purpureosericeum* of A contigs and 420 Mb of B contigs

	A contigs (bp)	*B contigs (bp)*
Genes	93,728,661	5,531,721
Tandem repeats	678,413	4,322,828
Ty1/copia	205,590,413	4,2254,642
Ty3/gypsy	443,738,910	91,104,319
Other LTRs	590,643,748	102,165,677
non-LTR	31,812,783	4,878,781
DNA transposons	46,689,765	6,605,022
Other sequences	992,953,798	163,042,214

Table S2: Primers for B-specific repeat clusters

B-repeat	*primer sequencies*	*annealing t (°C)*	*product size (bp)*
CL137_F<br>*CL137_R*	CGAGAGCCAACGTTCATTTT<br>TTAGCAATGGGATGGCTCTT	60	1125
CL166_F<br>*CL166_R*	CCTGTATCAAAATGTCTCCATGTC<br>ACTGCGTCCTAAAACGGTGA	60	344
CL193_F<br>*CL193_R*	CGAGAAAATGGAGCACAACC<br>AAGGGATGGTGCACTGGA	60	242
CL220_F<br>*CL220_R*	AAAACAATGGTCGGATGGAA<br>AAATGTAAGCTGCCAATTCTGA	60	1561

Table S3: B chromosome specific primers for candidate genes

candidate	*primer sequencies*	*annealing t (°C)*	*product size (bp)*
CENH3_F<br>*CENH3_R*	CTCTCCTTTCGTTTCGCCAT<br>AGCTTCTTCTTGGGCTTCTG	56	152
CENP-C_F<br>*CENP-C_R*	GCTTTTGTTGTGCATGACCA<br>GAGAACTCAAGCCATTCACACT	55	198
SMC3_F<br>*SMC3_R*	AGTGACTGGTTTGCTGGTTG<br>TTGTTGTTCCCAAGGTAGGTT	55	245
Naa50_F<br>*Naa50_R*	TGCGGGATAAGAACGAGCTC<br>AACAGAGAAGGGGAAGACGG	59	150
APC15_F<br>*APC15_R*	CTGTGATGGATCTCGTTGCG<br>ACTGCCTTCCAAGCCTAGAG	60	887
CYC1B_F<br>*CYC1B_R*	TAATGGAGGGTGGGGATT GC<br>TCATACAAGACTGACCTGGCT	58	153
