# Supplementary material for: Sorghum embryos undergoing B chromosome elimination express B-variants of mitotic-related genes
Source: Genome Biol. 2025 Dec 24;27:8. doi: 10.1186/s13059-025-03915-w (PMC12849586; doi:10.1186/s13059-025-03915-w)

Additional file 3: TopGO-directed hierarchical trees of all Gene Ontology (GO) terms identified through the GSEA analysis. Significant terms (p > 0.05) are depicted as boxes within the plot, with deeper colors representing greater levels of significance.

BP terms in early whole embryos

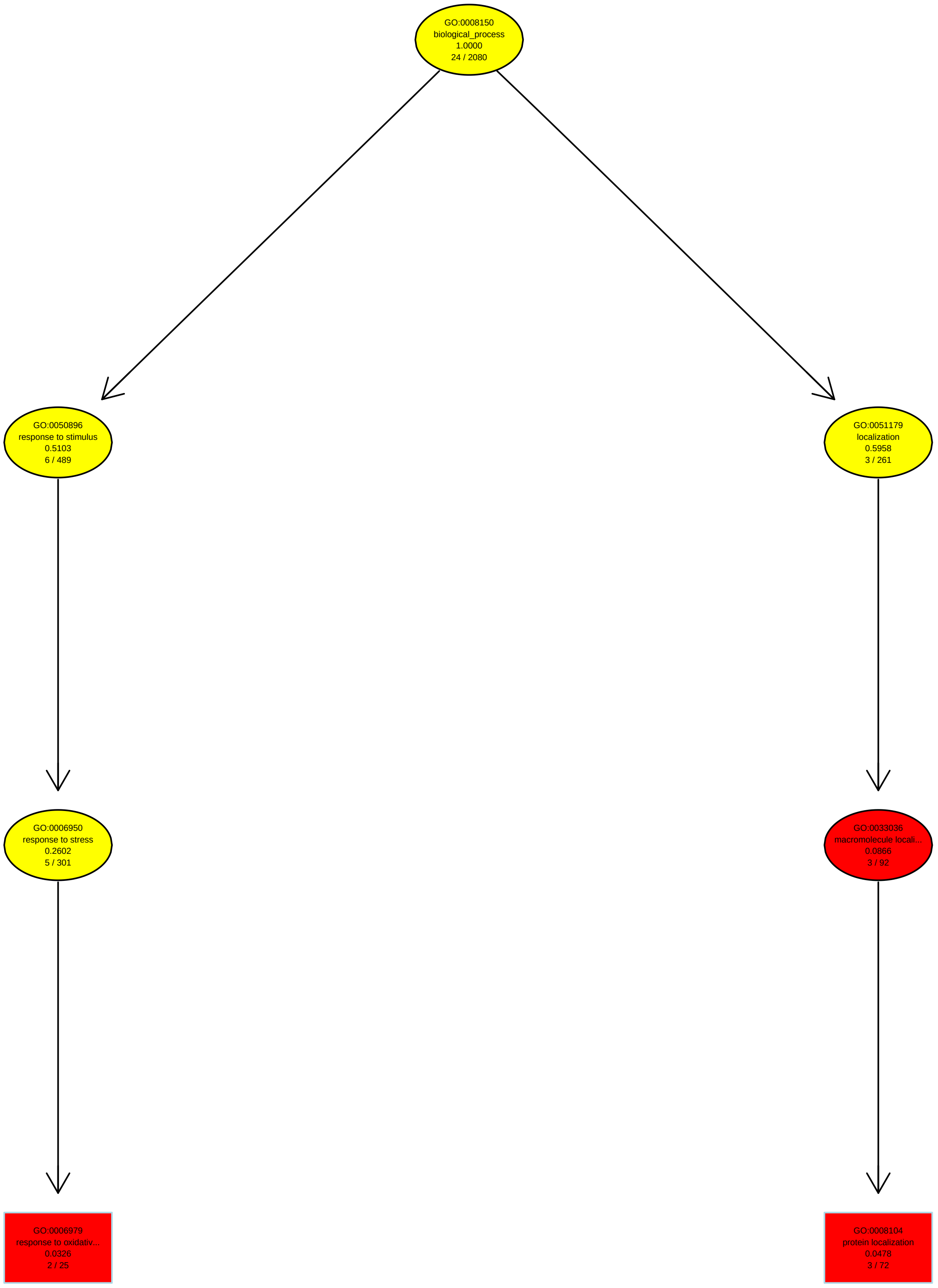

MF terms in early whole embryos

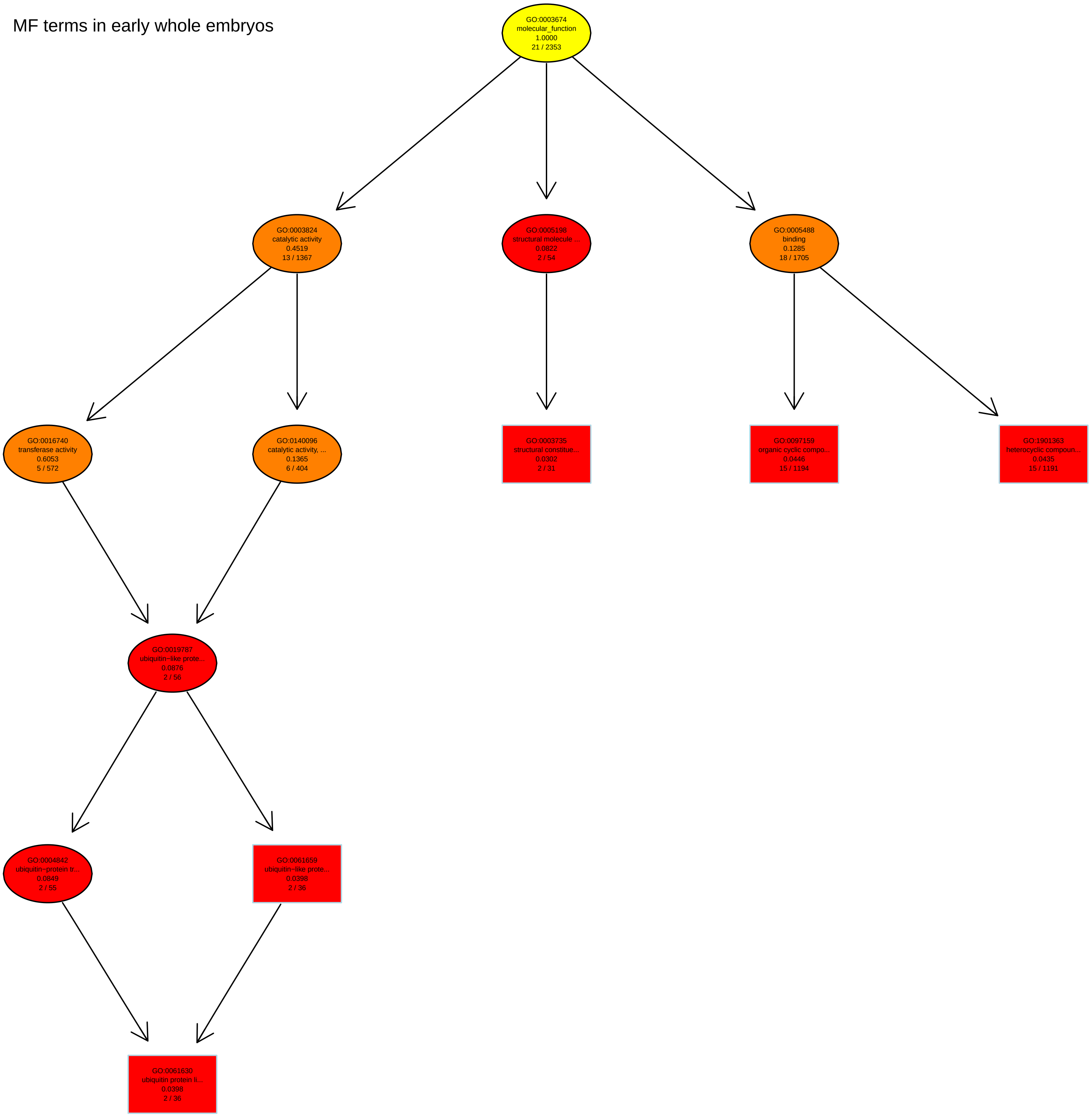

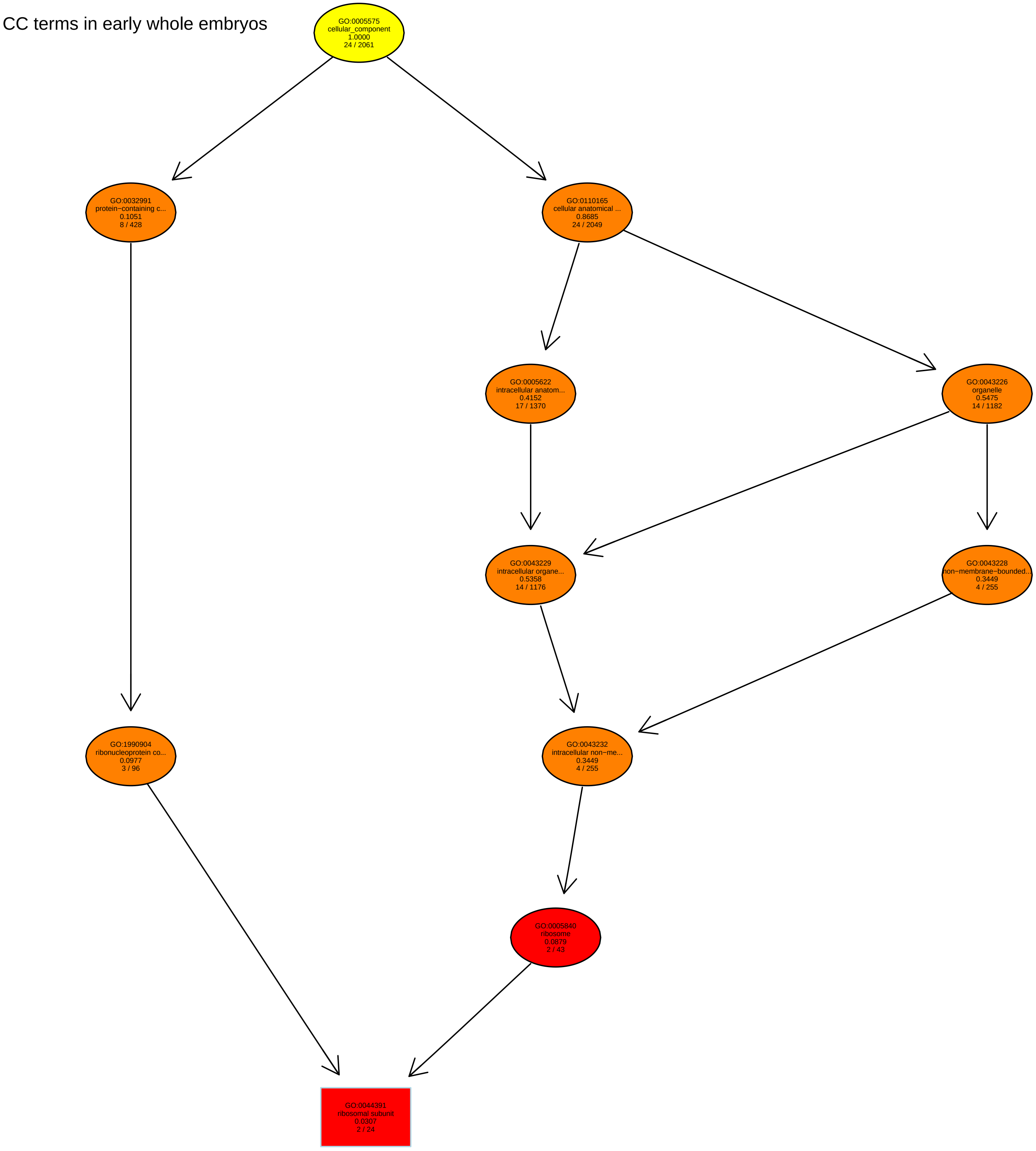
CC terms in early whole embryos
GO:0005575
cellular_component
1.0000
24 / 2061
GO:0032991
protein−containing c...
0.1051
8 / 428
GO:0110165
cellular anatomical ...
0.8685
24 / 2049
GO:0005622
intracellular anatom...
0.4152
17 / 1370
GO:0043226
organelle
0.5475
14 / 1182
GO:0043229
intracellular organe...
0.5358
14 / 1176
GO:0043228
non−membrane−bounded...
0.3449
4 / 255
GO:1990904
ribonucleoprotein co...
0.0977
3 / 96
GO:0043232
intracellular non−me...
0.3449
4 / 255
GO:0005840
ribosome
0.0879
2 / 43
GO:0044391
ribosomal subunit
0.0307
2 / 24

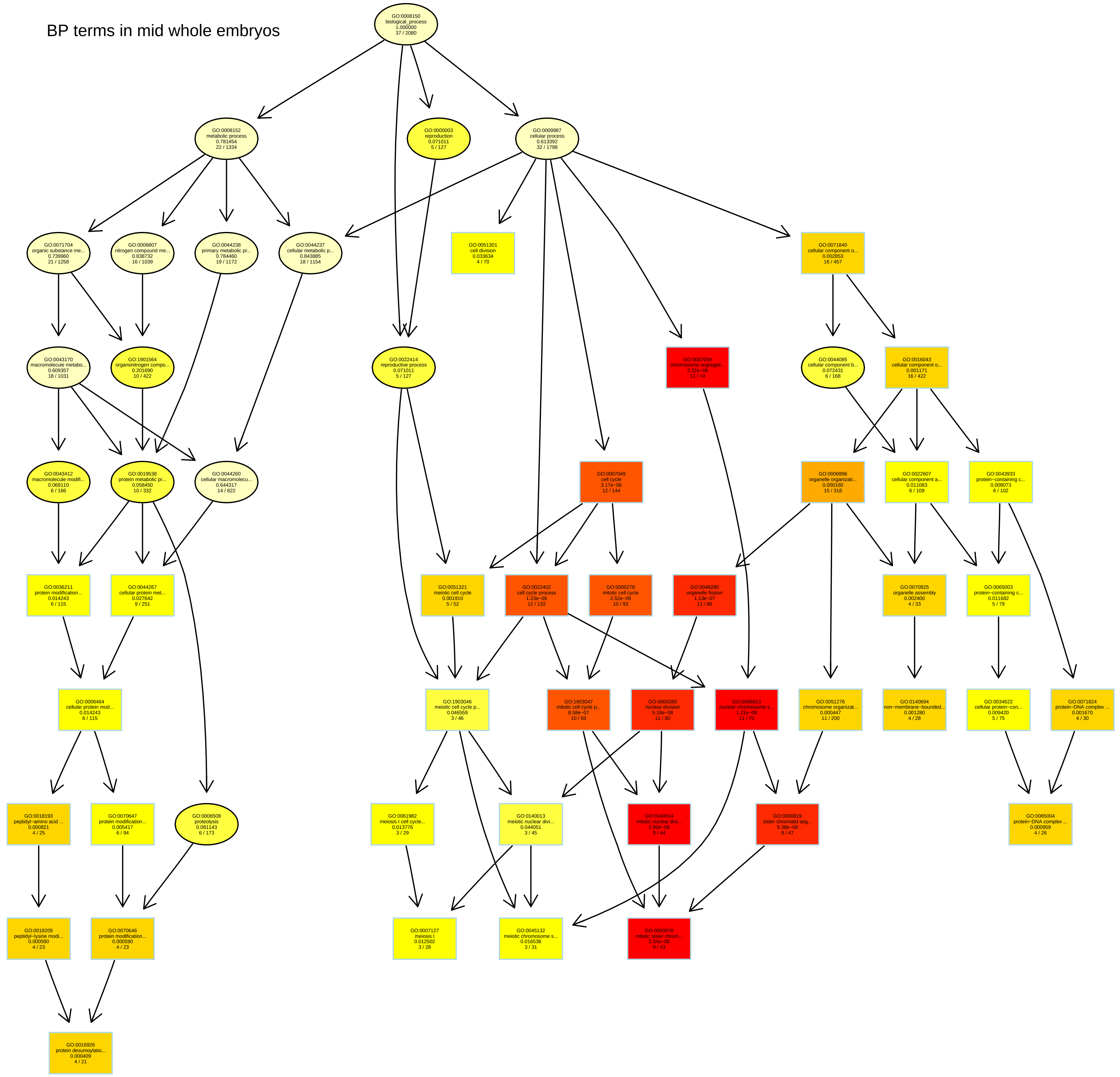

BP terms in mid whole embryos
GO:0008150 biological_process 1.000000 37 / 2080
GO:0008152 metabolic process 0.781454 22 / 1334
GO:0000003 reproduction 0.071011 5 / 127
GO:0009987 cellular process 0.613392 32 / 1798
GO:0071704 organic substance me... 0.739960 21 / 1258
GO:0006807 nitrogen compound me... 0.838732 16 / 1039
GO:0044238 primary metabolic pr... 0.784460 19 / 1172
GO:0044237 cellular metabolic p... 0.843885 18 / 1154
GO:0051301 cell division 0.033634 4 / 70
GO:0071840 cellular component o... 0.002853 16 / 457
GO:0043170 macromolecule metabo... 0.609357 18 / 1031
GO:1901564 organonitrogen compo... 0.201690 10 / 422
GO:0022414 reproductive process 0.071011 5 / 127
GO:0007059 chromosome segregati... 2.22e−08 11 / 74
GO:0044085 cellular component b... 0.072431 6 / 168
GO:0016043 cellular component o... 0.001171 16 / 422
GO:0043412 macromolecule modifi... 0.069110 6 / 166
GO:0019538 protein metabolic pr... 0.058450 10 / 332
GO:0044260 cellular macromolecu... 0.644317 14 / 822
GO:0007049 cell cycle 3.17e−06 12 / 144
GO:0006996 organelle organizati... 0.000160 15 / 318
GO:0022607 cellular component a... 0.011083 6 / 109
GO:0043933 protein−containing c... 0.008073 6 / 102
GO:0036211 protein modification... 0.014243 6 / 115
GO:0044267 cellular protein met... 0.027642 9 / 251
GO:0051321 meiotic cell cycle 0.001910 5 / 52
GO:0022402 cell cycle process 1.23e−06 12 / 132
GO:0000278 mitotic cell cycle 2.52e−06 10 / 93
GO:0048285 organelle fission 1.13e−07 11 / 86
GO:0070925 organelle assembly 0.002400 4 / 33
GO:0065003 protein−containing c... 0.011682 5 / 79
GO:0006464 cellular protein mod... 0.014243 6 / 115
GO:1903046 meiotic cell cycle p... 0.046559 3 / 46
GO:1903047 mitotic cell cycle p... 8.58e−07 10 / 83
GO:0000280 nuclear division 5.18e−08 11 / 80
GO:0098813 nuclear chromosome s... 1.21e−08 11 / 70
GO:0051276 chromosome organizat... 0.000447 11 / 200
GO:0140694 non−membrane−bounded... 0.001280 4 / 28
GO:0034622 cellular protein−con... 0.009420 5 / 75
GO:0071824 protein−DNA complex ... 0.001670 4 / 30
GO:0018193 peptidyl−amino acid ... 0.000821 4 / 25
GO:0070647 protein modification... 0.005417 6 / 94
GO:0006508 proteolysis 0.081143 6 / 173
GO:0061982 meiosis I cell cycle... 0.013776 3 / 29
GO:0140013 meiotic nuclear divi... 0.044051 3 / 45
GO:0140014 mitotic nuclear divi... 2.90e−08 9 / 44
GO:0000819 sister chromatid seg... 5.38e−08 9 / 47
GO:0065004 protein−DNA complex ... 0.000959 4 / 26
GO:0018205 peptidyl−lysine modi... 0.000590 4 / 23
GO:0070646 protein modification... 0.000590 4 / 23
GO:0007127 meiosis I 0.012502 3 / 28
GO:0045132 meiotic chromosome s... 0.016538 3 / 31
GO:0000070 mitotic sister chrom... 2.34e−08 9 / 43
GO:0016926 protein desumoylatio... 0.000409 4 / 21

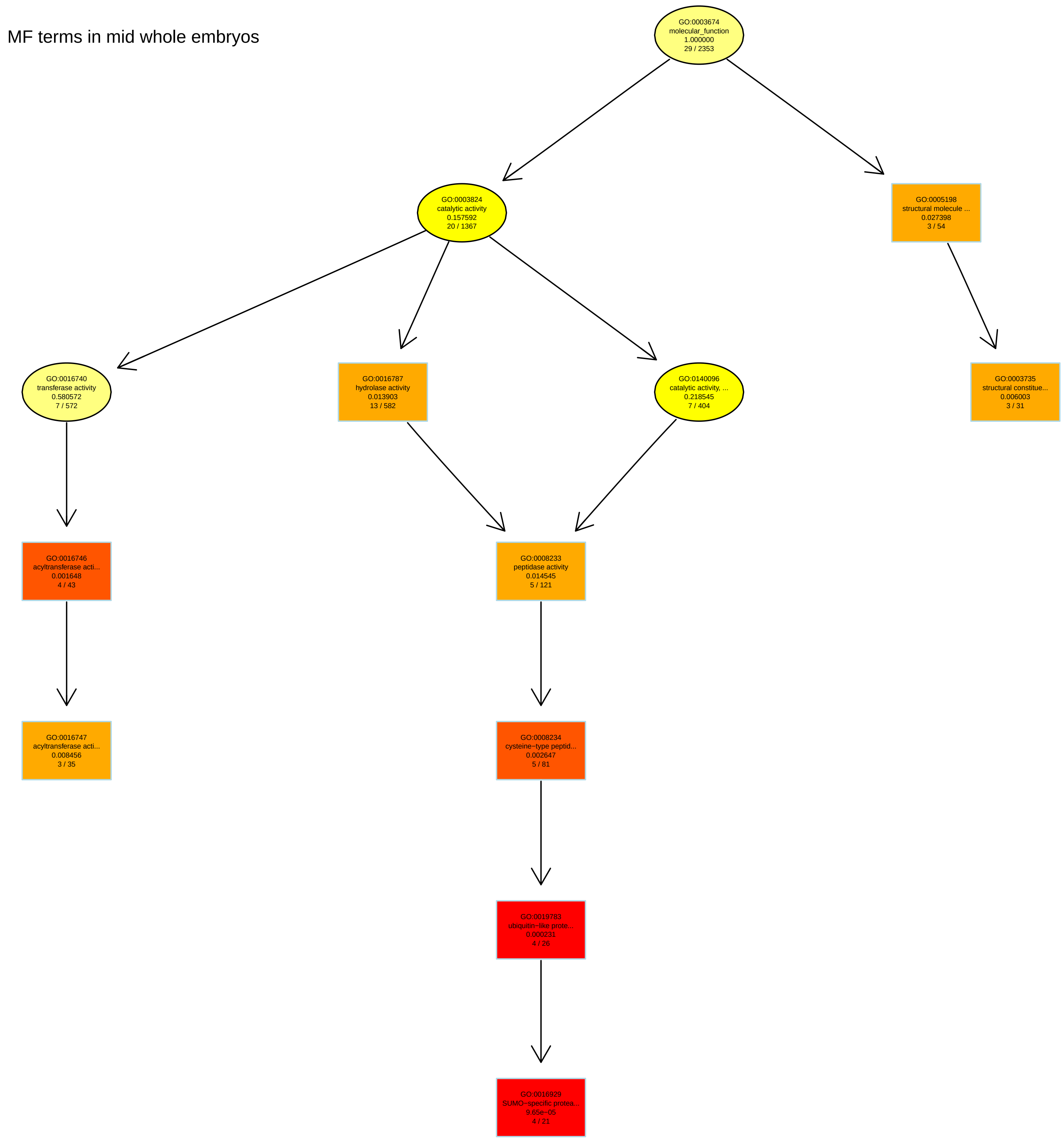
MF terms in mid whole embryos
GO:0003674 molecular_function 1.000000 29 / 2353
GO:0003824 catalytic activity 0.157592 20 / 1367
GO:0005198 structural molecule ... 0.027398 3 / 54
GO:0016740 transferase activity 0.580572 7 / 572
GO:0016787 hydrolase activity 0.013903 13 / 582
GO:0140096 catalytic activity, ... 0.218545 7 / 404
GO:0003735 structural constitue... 0.006003 3 / 31
GO:0016746 acyltransferase acti... 0.001648 4 / 43
GO:0008233 peptidase activity 0.014545 5 / 121
GO:0016747 acyltransferase acti... 0.008456 3 / 35
GO:0008234 cysteine−type peptid... 0.002647 5 / 81
GO:0019783 ubiquitin−like prote... 0.000231 4 / 26
GO:0016929 SUMO−specific protea... 9.65e−05 4 / 21

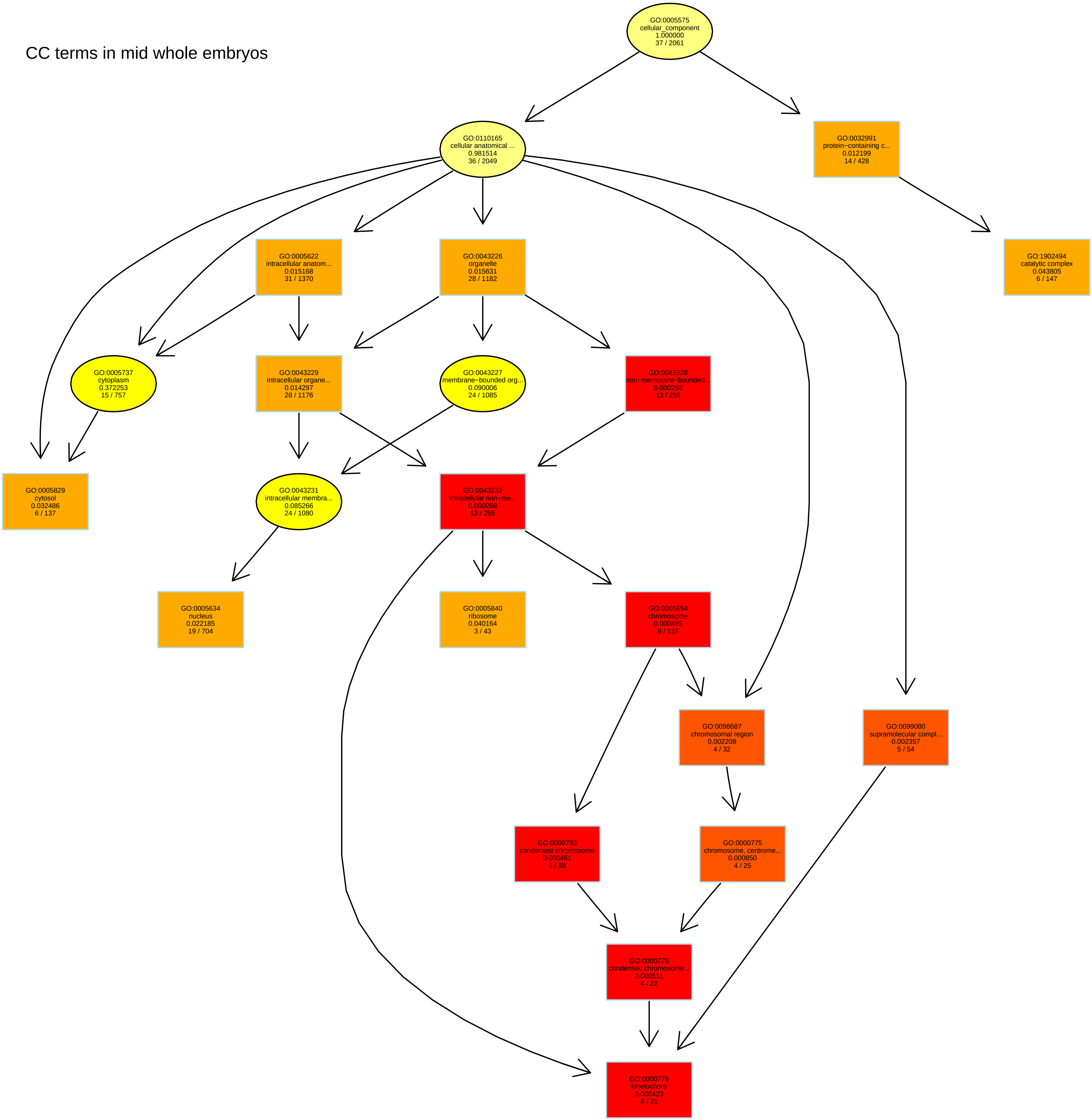
CC terms in mid whole embryos
GO:0005575
cellular_component
1.000000
37 / 2061
GO:0110165
cellular anatomical ...
0.981514
36 / 2049
GO:0032991
protein−containing c...
0.012199
14 / 428
GO:0005622
intracellular anatom...
0.015168
31 / 1370
GO:0043226
organelle
0.015631
28 / 1182
GO:1902494
catalytic complex
0.043805
6 / 147
GO:0005737
cytoplasm
0.372253
15 / 757
GO:0043229
intracellular organe...
0.014297
28 / 1176
GO:0043227
membrane−bounded org...
0.090006
24 / 1085
GO:0043228
non−membrane−bounded...
0.000268
13 / 255
GO:0005829
cytosol
0.032486
6 / 137
GO:0043231
intracellular membra...
0.085266
24 / 1080
GO:0043232
intracellular non−me...
0.000268
13 / 255
GO:0005634
nucleus
0.022185
19 / 704
GO:0005840
ribosome
0.040164
3 / 43
GO:0005694
chromosome
0.000489
9 / 137
GO:0098687
chromosomal region
0.002208
4 / 32
GO:0099080
supramolecular compl...
0.002357
5 / 54
GO:0000793
condensed chromosome
0.000461
5 / 38
GO:0000775
chromosome, centrome...
0.000850
4 / 25
GO:0000779
condensed chromosome...
0.000511
4 / 22
GO:0000776
kinetochore
0.000423
4 / 21

BP terms in late whole embryos

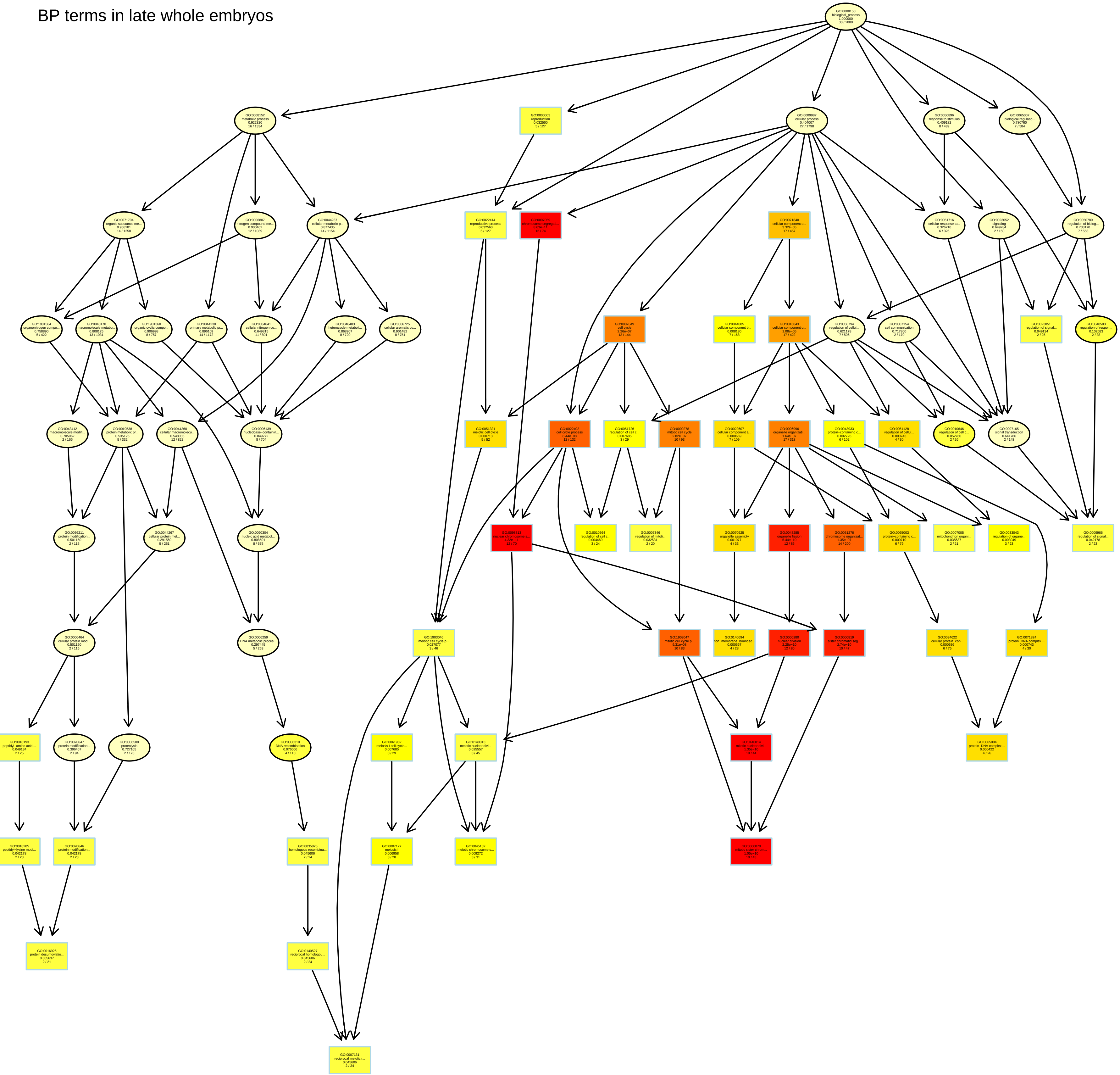

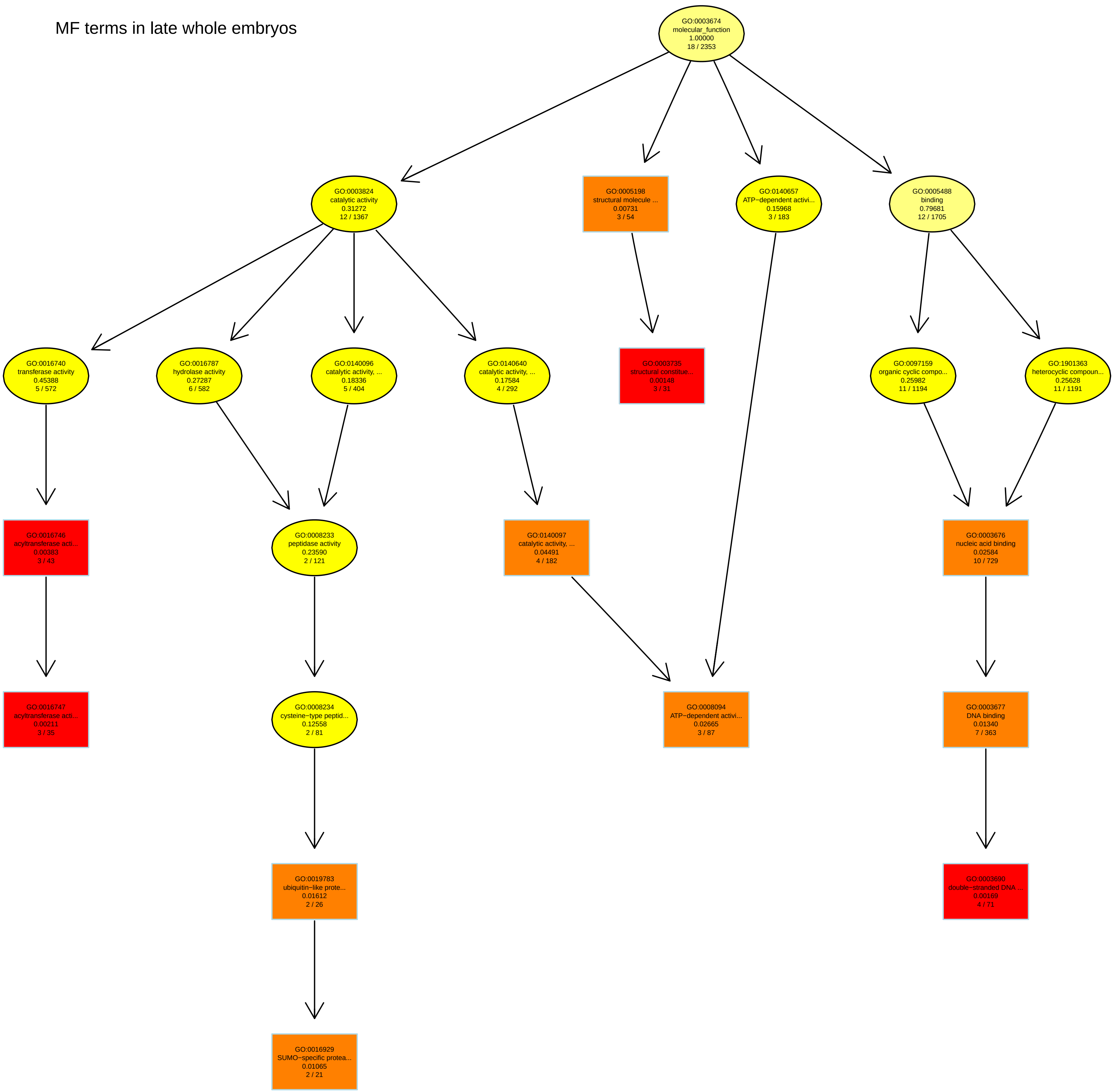
MF terms in late whole embryos
GO:0003674
molecular_function
1.00000
18 / 2353
GO:0003824
catalytic activity
0.31272
12 / 1367
GO:0005198
structural molecule ...
0.00731
3 / 54
GO:0140657
ATP−dependent activi...
0.15968
3 / 183
GO:0005488
binding
0.79681
12 / 1705
GO:0016740
transferase activity
0.45388
5 / 572
GO:0016787
hydrolase activity
0.27287
6 / 582
GO:0140096
catalytic activity, ...
0.18336
5 / 404
GO:0140640
catalytic activity, ...
0.17584
4 / 292
GO:0003735
structural constitue...
0.00148
3 / 31
GO:0097159
organic cyclic compo...
0.25982
11 / 1194
GO:1901363
heterocyclic compoun...
0.25628
11 / 1191
GO:0016746
acyltransferase acti...
0.00383
3 / 43
GO:0008233
peptidase activity
0.23590
2 / 121
GO:0140097
catalytic activity, ...
0.04491
4 / 182
GO:0003676
nucleic acid binding
0.02584
10 / 729
GO:0016747
acyltransferase acti...
0.00211
3 / 35
GO:0008234
cysteine−type peptid...
0.12558
2 / 81
GO:0008094
ATP−dependent activi...
0.02665
3 / 87
GO:0003677
DNA binding
0.01340
7 / 363
GO:0019783
ubiquitin−like prote...
0.01612
2 / 26
GO:0003690
double−stranded DNA ...
0.00169
4 / 71
GO:0016929
SUMO−specific protea...
0.01065
2 / 21

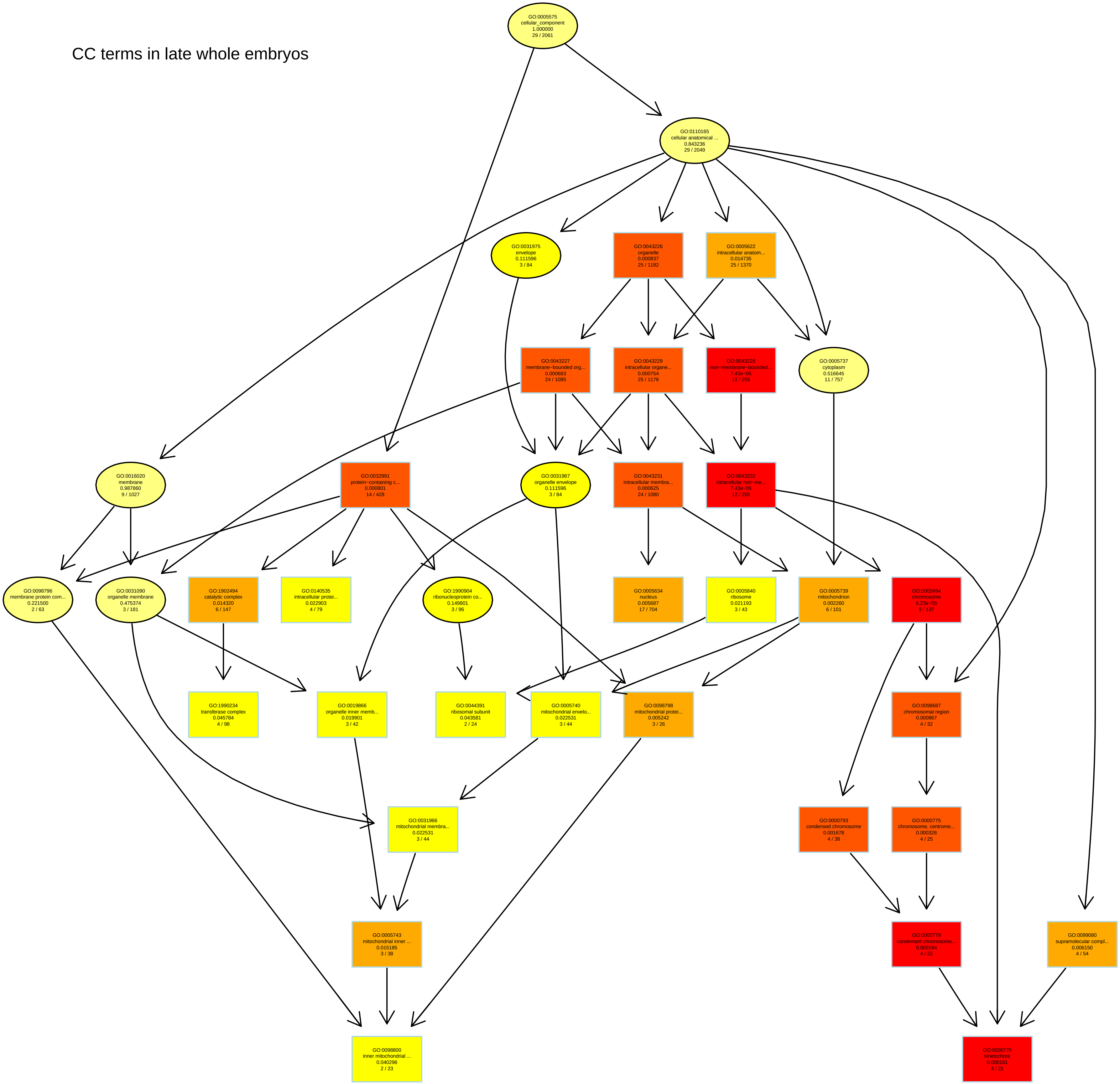
CC terms in late whole embryos
GO:0005575
cellular_component
1.000000
29 / 2061
GO:0110165
cellular anatomical ...
0.843236
29 / 2049
GO:0031975
envelope
0.111596
3 / 84
GO:0043226
organelle
0.000837
25 / 1182
GO:0005622
intracellular anatom...
0.014735
25 / 1370
GO:0043227
membrane−bounded org...
0.000683
24 / 1085
GO:0043229
intracellular organe...
0.000754
25 / 1176
GO:0043228
non−membrane−bounded...
7.43e−05
12 / 255
GO:0005737
cytoplasm
0.516645
11 / 757
GO:0016020
membrane
0.987860
9 / 1027
GO:0032991
protein−containing c...
0.000801
14 / 428
GO:0031967
organelle envelope
0.111596
3 / 84
GO:0043231
intracellular membra...
0.000625
24 / 1080
GO:0043232
intracellular non−me...
7.43e−05
12 / 255
GO:0098796
membrane protein com...
0.221500
2 / 63
GO:0031090
organelle membrane
0.475374
3 / 181
GO:1902494
catalytic complex
0.014320
6 / 147
GO:0140535
intracellular protei...
0.022903
4 / 79
GO:1990904
ribonucleoprotein co...
0.149801
3 / 96
GO:0005634
nucleus
0.005687
17 / 704
GO:0005840
ribosome
0.021193
3 / 43
GO:0005739
mitochondrion
0.002260
6 / 101
GO:0005694
chromosome
6.23e−05
9 / 137
GO:1990234
transferase complex
0.045784
4 / 98
GO:0019866
organelle inner memb...
0.019901
3 / 42
GO:0044391
ribosomal subunit
0.043581
2 / 24
GO:0005740
mitochondrial envelo...
0.022531
3 / 44
GO:0098798
mitochondrial protei...
0.005242
3 / 26
GO:0098687
chromosomal region
0.000867
4 / 32
GO:0031966
mitochondrial membra...
0.022531
3 / 44
GO:0000793
condensed chromosome
0.001678
4 / 38
GO:0000775
chromosome, centrome...
0.000326
4 / 25
GO:0005743
mitochondrial inner ...
0.015185
3 / 38
GO:0000779
condensed chromosome...
0.000194
4 / 22
GO:0099080
supramolecular compl...
0.006150
4 / 54
GO:0098800
inner mitochondrial ...
0.040296
2 / 23
GO:0000776
kinetochore
0.000161
4 / 21

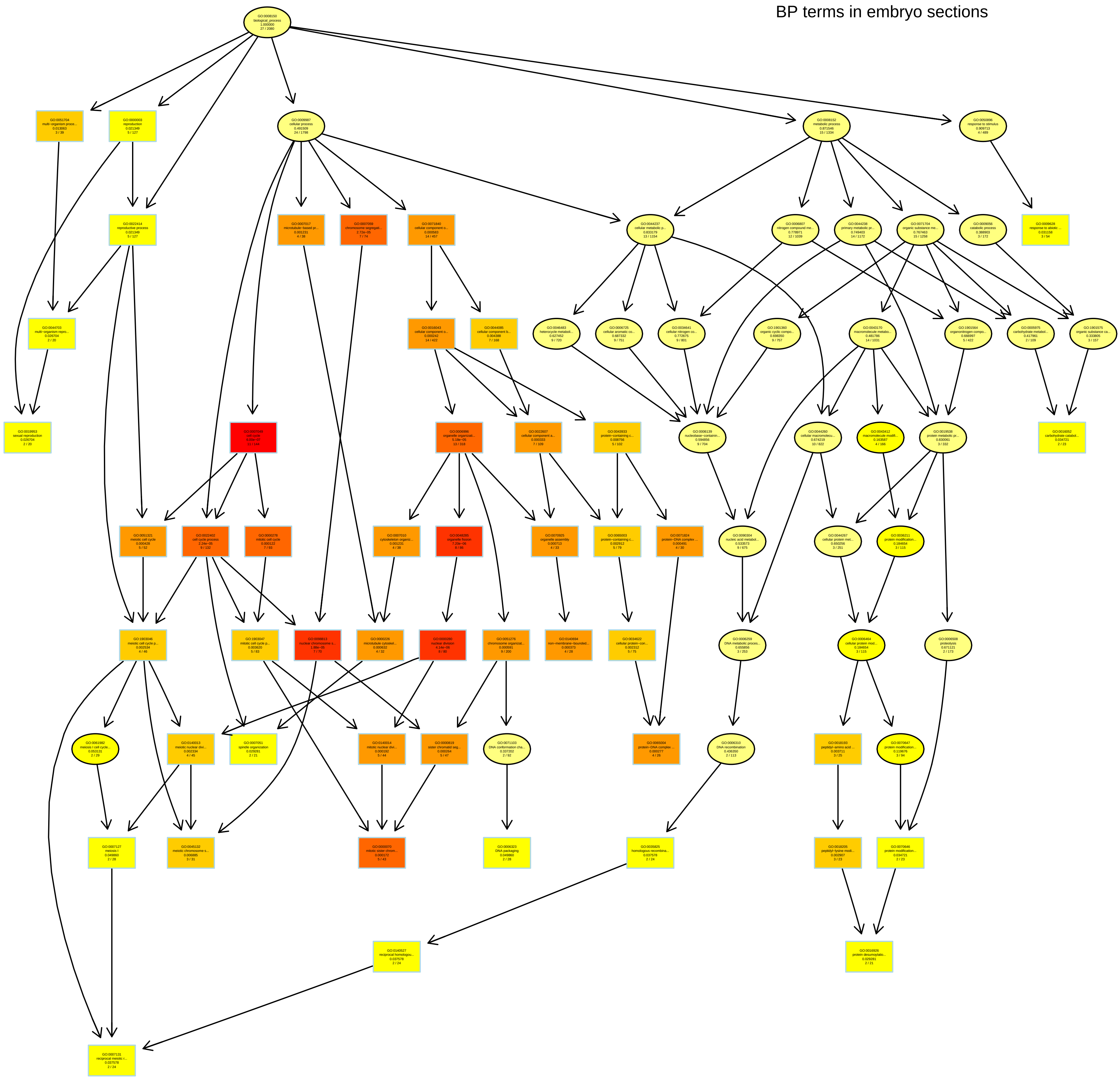
BP terms in embryo sections

MF terms in embryo sections

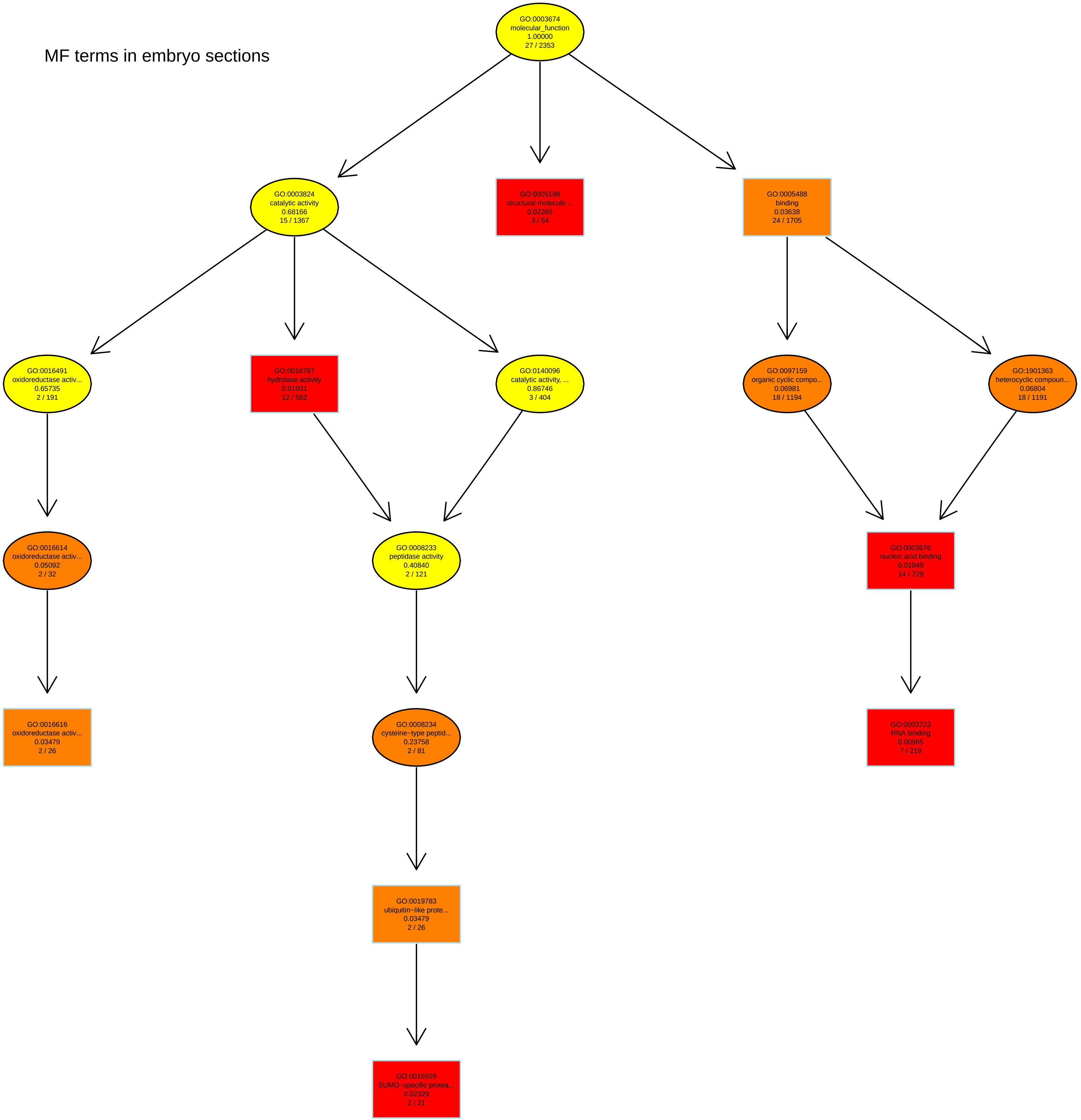

CC terms in embryo sections

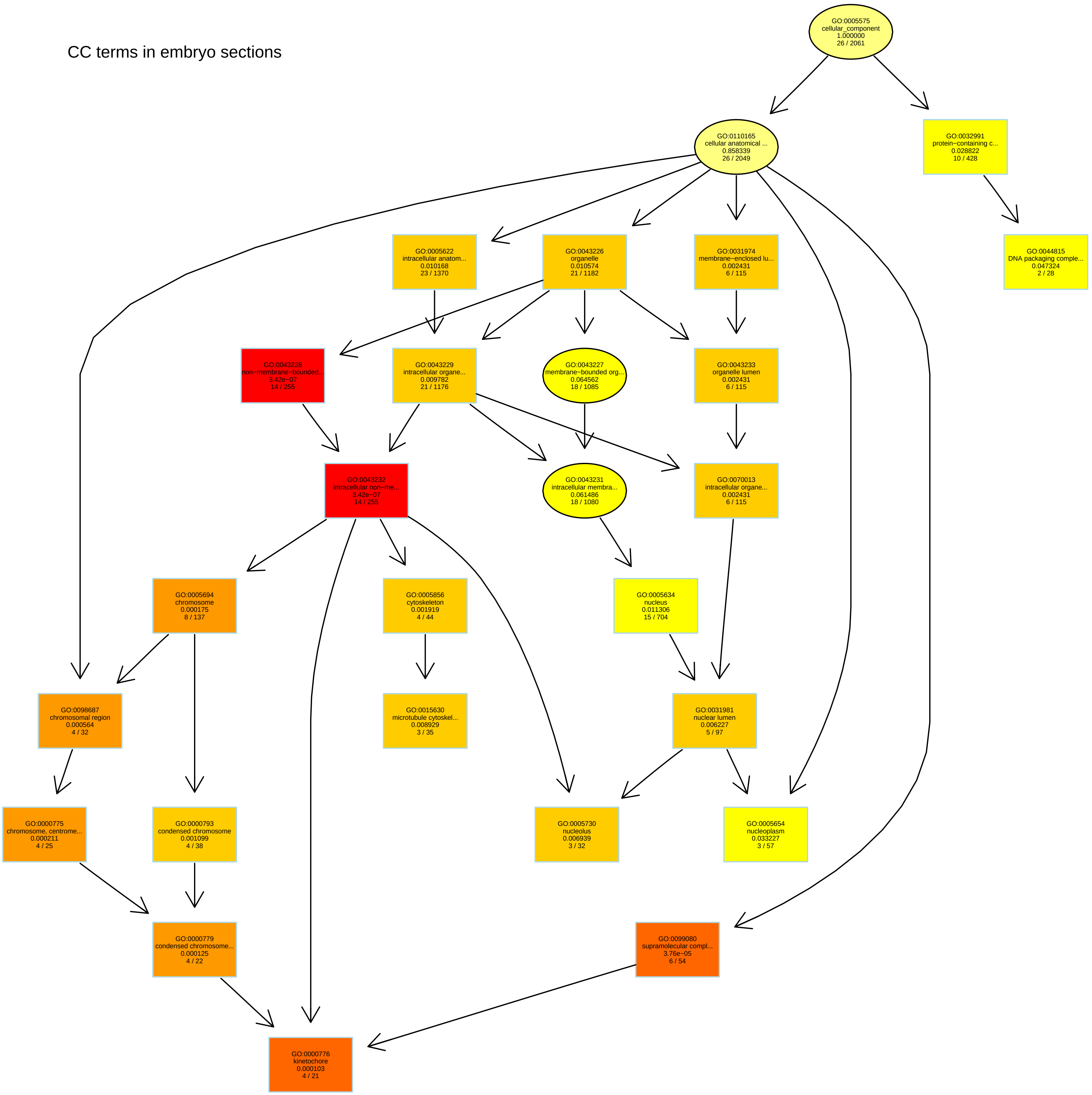

Supplement: Supplementary file 3 — Additional file 3. Supplementary Document (file contains hierarchy trees of all significant GO terms). [file 13059_2025_3915_MOESM3_ESM.pdf]
