# Supplementary material for: Sorghum embryos undergoing B chromosome elimination express B-variants of mitotic-related genes
Source: Genome Biol. 2025 Dec 24;27:8. doi: 10.1186/s13059-025-03915-w (PMC12849586; doi:10.1186/s13059-025-03915-w)
Supplement: Supplementary file 4 — Additional file 4. Supplementary Document (file contains alignments of A/B chromosome variants of candidate genes). [file 13059_2025_3915_MOESM4_ESM.pdf]

Additional file 4: Alignments of A/B chromosome variants of candidate genes. Alignments were obtained using EMBOSS Needle Pairwise Sequence Alignment tool. An asterisk (*) indicates positions with identical residues in both sequences, a colon (:) marks positions with conserved substitutions, and a period (.) signifies positions with semi-conserved substitutions.

CENH3 – 98.7% identity

```
CENH3_A,g22204       MARTKHQAVRRPTQKPKKKLQLERGGAGTSATPERNAGAGEGTAARGARGRGEKKKMRWR     60
CENH3_B,g22416       MARTKHQAVRRPTQKPKKKLQLERGGAGTSATPERNAGAGEGTAARGARGRGEKKKMRWR     60
                     ************************************************************

CENH3_A,g22204       PGTVALREIRRYQKSTEPLIPFAPFVRVVKEITGFVTYWRIGRYTPEALLALQEAAEFHL     120
CENH3_B,g22416       PGTVALREIRRYQKSTEPLIPFAPFVRVVKEVTGFVTYWRIGRYTPEALLALQEAAEFHL     120
                     *******************************:****************************

CENH3_A,g22204       IELFEVANLCAIHAKRVTVMQKDIQLARRIGGRRWS*   156
CENH3_B,g22416       IELFEVANLCAIHAKRVTVMQKDMQLARRIGGRRWS*   156
                     ***********************:*************
```

CENP-C – 74.32% identity

```
CENP-C_A,g28463      MDVADPLCAISSPARLLPRTLGPAPASSSSSSSSTGLLEAIAVARSLKGSEELLKQAKMV     60
CENP-C_B,g5138       ------------------------------------------------------------     0

CENP-C_A,g28463      LKEHGDIQALYPDDGVQARPPVNGSKEQQGRRPALNRKRSRFTMKETASKPMPVVDRSKL     120
CENP-C_B,g5138       ---------------------------------------------------MPVVDRSKL     9
                                                                        *********

CENP-C_A,g28463      TNISDPVEFFMTLDRLD--EAEEEILRLTGAAEKRVLNFDPVDEPKRQPGFRG---RKSV     175
CENP-C_B,g5138       TNTSDPFKNFKTVDPLDIAEAEEEILRLSGAAEKRVLNFDPVDEPKRQPGFRGYASRKSV     69
                     ** ***.: * *:* **  *********:************************   ****

CENP-C_A,g28463      CSFRIIEDADTQDPLEVPASQTGSQFPQDVMHVVADKNERVPSSSDEAISGKEDSLAEKD     235
CENP-C_B,g5138       CSFRTNEDADTQDPLEVPASQTGSQFPQDVMHVVADKNERVPSSSGEAISGKE-------     122
                     ****  ***************************************.*******

CENP-C_A,g28463      GRDDLTYLLTSMQHLDESKEEEFIRKTLGVKDIRKERVSLRNSIPGVRPLRTEREVSMRV     295
CENP-C_B,g5138       -FDDLTYLLTSMQHLDESEEEEFIRKTLGVKEIRKERVSL-------RPLRTEREVSMRV     174
                       ****************:************:********       *************

CENP-C_A,g28463      HPPESPLPQPLQDRISELEKHLFHEDAANAKCTDDESEGSPDIVMGEPSLVHDSSDVPMT     355
CENP-C_B,g5138       HPPESSLPQPLQDQISELEKHLFHEDAANAECTDDEYEGSPDIVMGEPSLVHDSSDVPMT     234
                     ***** *******:****************:***** ***********************

CENP-C_A,g28463      DENSTVSEIDRDTPNLGARAADHILDPEPDMPDHAYERQPGDSSVGLCRDTQVAKENEAC     415
CENP-C_B,g5138       DENSTVSEIHRDTPNLGARAADHILDPEPDTPDHAYERQPGGSSVGLCRDTEVTKENEAW     294
                     *********.******************** **********.*********:*:*****

CENP-C_A,g28463      RRSNISV----------EEDDVPIDHPTIGRSTSETEASSHHLERSSTEELVNKPGRHGA     465
CENP-C_B,g5138       RRSNISVEACISIYFALQEDDVPIDHPTIGRSTSETEASSHHLEGSSTEELVNKPGRHGA     354
                     *******          :************************** ***************

CENP-C_A,g28463      PDGINRTLHAAEDIIQHLEVVKEGGVLQDKSSQSLEMPLEDIDPVNQPQMHGGSTKKLAP     525
CENP-C_B,g5138       PDGIDSTLHAAEDIIQHLST--------DKSSQSLEMPLEDIDPVNQPQMHGGSTKKLAP     406
                     ****: ************..        ********************************

CENP-C_A,g28463      DVCNALSLTKQKKQQAAQEGKMKRQSKRGKKVADESSHVLEIPQANLDSENQPHNDDVNI     585
CENP-C_B,g5138       DLCNALSLTKRKKQQAAQAGKMKRQSKRGKKVADEPSHVLEIPKANLDNDDVNIEKSAHD     466
                     *:********:******* **************** *******:****.::   :...:

CENP-C_A,g28463      EQQTVLSITP-----------------------SPNHA----------------------     600
CENP-C_B,g5138       -SCNALSLTKQKQQAAQEGKMKKQPKRGKKVADEPSHVLEIPKANLDNDDVNIEKSAHDS     525
                      . ..**:*                        .*.*.

CENP-C_A,g28463      --------EGQKGAQITNKTKKMNQRKILGDGGLAQPSVVRRSTRTRSRPLEHWLGERLL     652
CENP-C_B,g5138       CNALSLTKQKQQAAQEGKMKKQPKRGKKVADGGVAQP--LRRSTRTRSRPLKHWLGERLL     583
                             : *:.**  : .*: :: * :.***:***  :***********:********
```

```
CENP-C_A,g28463      YGPINDTLPAVIGIKAYSPDQDGKRTLKVKSFVPDQFSDLVAKSAKY*     699
CENP-C_B,g5138       YGPINDTLPAVIGIKTYSPDQDGKRTLKVESFVPDQFSDLIAKSAKY*     630
                     ***************:*************:**********:*******
```

Mis12 – 81.37% identity

```
Mis12_B,g14525       MEDCDESAAT-AAEAALGLNPQHFFNEVHGIIADISAGAFE----AAAAPGVVGAAKAAE      55
Mis12_A,g32810       MEDCDESAAAAAVEAALGLNPQLFVNEVHGIIADIGAGAFEYGLQAAAAPGVVGAAKAAE      60
                     *********: *.********* *.**********.*****    ***************

Mis12_B,g14525       KATDLQRGLNAIHHVVKNRLDKRMTNWKAFCLRHCFDVPEGFVAAEDDRC-AKESHKDET      114
Mis12_A,g32810       KATDLQRGLNAIHHVVKGRLDKRMTNWKAFCLRHCFDVPEGFVAAEDDRSRAKESHKDET      120
                     *****************.*******************************. *********

Mis12_B,g14525       SGLNLELDSLRRKLESATKESQNLEREMSSLERQTTCKRQLDSSLSEIQKLFKDKSVQEN      174
Mis12_A,g32810       SDLDLELDSLRRKLESANKESQNLEREISSLERQTTYKRQLDSSLSEIQKLFEEKSVQEN      180
                     *.*:*************.*********:******** ***************::******

Mis12_B,g14525       FEGVVKAASVLKQKIIDMKKKRTATTCSDQSVWNTNNLTDNRRQTLDNGFTACAEDIQET      234
Mis12_A,g32810       FEGLLKAVPVLKQKIIDLNKKRTATTCSDQQVWNTNNLTDNRRQTLDN-------DIQET      233
                     ***::**. ********::***********.*****************       *****

Mis12_B,g14525       ISILKNKCHVGPLSLPQGAQDQGRRHLDGTSSSNIPGISKKA-RVKGIKEGKNQGI*   289
Mis12_A,g32810       ISIVKNKCRGAVI-TPTGSAGPGPQ---------APGWHQQQQHSRDQQEGKN*---   276
                     ***:****: . :  * *: . * :          **  ::  : :. :****
```

Nuf2 – 89.47% identity

```
Nuf2_B,g8044        MAMQKVQEKTNTLEMYTKFSEKLANHLSKISAVLEKSAAAKASEKDVKAHKEKISDQNLE 60
Nuf2_A,g53293       MAMQKVQEKTNTLEVYTKVSEKLAKHLSKISTVLEKSAAAKASEKDVKAHKEKISDQNLE 60
                    **************:***.*****:******:****************************

Nuf2_B,g8044        IKALRNKAAEWQMRVLENEAKLKAKEKERDQRVGENNRKMTALKSEVELEHKCLEERQRK 120
Nuf2_A,g53293       IKALRNKAAEWQMKVLENEAKLKAKE-ERDQRVGENKRKMAALKSEVESEHKCLEEKQRK 119
                    *************:************ *********:***:******* *******:***

Nuf2_B,g8044        IKEKIDKGSELCSQADSVAEAGWKKIEEIYAKFDQVCEAAKVYMDGMDQSFDETDEAAVT 180
Nuf2_A,g53293       IKEKIDKGSELCSQADSVAEAGRKKIEEIHGKFDQVSEAAKMYVDGMDQSFDETDEDAVM 179
                    ********************** ******:.*****.****:*:************ **

Nuf2_B,g8044        LSTVARGGA*    189
Nuf2_A,g53293       LSTIARNGA*    188
                    ***:**.***
```

Naa50 – 98.36% identity

```
Naa50_A,g12466       MGAGDGEVAASKEKGSGGAGGGGVERTSLDGVRDKNVMQLKKLNTALFPVRYNEKYYQDA      60
Naa50_B,g13475       MGAGDGEVAASKEKGSGGAGGGGVERTSLDGVRDKNE--LKKLNTALFPVRYNEKYYQDA      58
                     ************************************   *********************

Naa50_A,g12466       IASKDFSKLAYYSDICVGAIACRLEKKEGGAIRVYIMTLGVLAPYRGLGIGTKLLNHVFD      120
Naa50_B,g13475       IASKDFSKLAYYSDICVGAIACRLEKKEGGAIRVYIMTLGVLAPYRGLGIGTKLLNHVFD      118
                     ************************************************************

Naa50_A,g12466       LSAKQNISEIYLHVQTNNDDAIAFYKKFGFEITQTIHNYYMNITPPDCYVLTKFIGQAAT      180
Naa50_B,g13475       LSAKQNISEIYLHVQTNNDDAIAFYKKFGFEITQTIHNYYMNITPPDCYVLTKFIGQAAT      178
                     ************************************************************

Naa50_A,g12466       KK*   182
Naa50_B,g13475       KK*   180
                     ***
```

SMC3 – 99.01% identity

```
SMC3_A,g38923      MYIKKVIIEGFKSYREEISTEPFSPKVNVVVGANGSGKSNFFHAIRFVLSDMFQNLRSED 60
SMC3_B,g36126      MYIKKVIIEGFKSYREEISTEPFSPKVNVVVGANGSGKSNFFHAIRFVLSDMFQNLRSED 60
                   ************************************************************

SMC3_A,g38923      RGALLHEGAGHSVVSAFVEIVFDNSDNRIPVDKEEVRLRRTVASKKDEYYLDGKHVSKTE 120
SMC3_B,g36126      RGALLHEGAGHSVVSAFVEIVFYNSDNRIPVDKEEVRLRRTVASKKDEYYLDGKHVSKTE 120
                   ********************** *************************************

SMC3_A,g38923      VMNLLESAGFSRSNPYYVVQQGKIASLTLMKDSERLDLLKEIGGTRVYEDRRKESLKIMT 180
SMC3_B,g36126      VMNLLESAGFSRSNPYYVVQQGKIASLTLMKDSERLDLLKEIGGTRVYEDRRKESLKIMT 180
                   ************************************************************

SMC3_A,g38923      ETANKRKQIDQVVHYLEERLRELDEEKDELKKYQQLDKQKRSLEYTILDHELNDARNELA 240
SMC3_B,g36126      ETANKRKQIGQVVHYLEERLRELDEEKDELKKYQQLDKQKRSLEYTILDHELNDARNELA 240
                   *********.**************************************************

SMC3_A,g38923      SMDDNRRKISESMSLADNEVVDVREMIKSFDKEIKVSTKGINDTKAQKEGVEKRRTEALK 300
SMC3_B,g36126      SMDDNRRKISESMSLADNEVVDVREMIKSFDKEIKVSTKGINDTKAQKEGVEKRHTEALK 300
                   ******************************************************:*****

SMC3_A,g38923      VVAQIELDLRDIKDRIVNEKRAKDEAARDLQSVRRESEKSKSELAEISKVHLTKLKEEEE 360
SMC3_B,g36126      VVAQIELDLRDIKDRIVNEKRAKDEAARDLQSVRRESEKSKSELAEISKVHQTKLKEEEE 360
                   *************************************************** ********

SMC3_A,g38923      ISKSIMDREKRLSILYQKQGRATQFANKAARDKWLQKEIEDLKPVLLSNRKQEGLLQEEI 420
SMC3_B,g36126      ISKSIMDREKRLSILYQKQGRATQFANKAARDKWLQKEIEDLKPVLLSNRKQEGLLQEEI 420
                   ************************************************************

SMC3_A,g38923      QKLKDDITELTNYIESRKNESSKLEEALAKRHNDYNDLRKQRDVLQEERKSYWKEESEVT 480
SMC3_B,g36126      QKLKDDITELTNYIESRKNESSKLEEALAKRHNDYNDLRKQRDVLQEERKSYWKEESEVT 480
                   ************************************************************

SMC3_A,g38923      AELDRLQEELVKAQKSLDHATPGDIRRGLTSVNNIIKECSITGVFGPVLELIDCEEKFFT 540
SMC3_B,g36126      AELDRLQEELVKAQKSLDHATPGDIRRGLTSVNNIIKECSITGVFGPVLELIDCEEKFFT 540
                   ************************************************************

SMC3_A,g38923      AVEVTAGNSLFHVVVENDDISTRIIEHLNKRKGGRVTFIPLNRVKASDLSCPQSPDFVPL 600
SMC3_B,g36126      AVEVTAGNSLFHVVVENDDISTRIIEHLNKRKGGRVTFIPLNRVKAPDLSCPQSPDFVPL 600
                   ********************************************** *************

SMC3_A,g38923      LKKLKYRAEHRRAFEQVFGRTVICRDLETATKVARSNGLDCITLDGDQVGKKGAMTGGFY 660
SMC3_B,g36126      LKKLKYRAEHRRAFEQVFGRTVICRDLETATKVARSNGLDCITLDGDQVGKKGAMTGGFY 660
                   ************************************************************

SMC3_A,g38923      DSRRSKLKLVKIFRDNKTAIEKKATHLEAVGNKLKDIDKKITDLVTKQQQMDAERDHAKL 720
SMC3_B,g36126      DSRRSKLRLVKIFRDNKTAIEKKATHLEAVGNKLKDIDKKITDLVTKQQQMDAERDHAKL 720
                   *******:****************************************************

SMC3_A,g38923      ELEQFKVDIARAMKQKASLEKALGKKEKSLDNIRNQIEQVQSSIAMKNDEMGTELIDQLT 780
SMC3_B,g36126      ELEQFKVDIARAMKQKASLEKALGKKEKSLDNIRNQIEQVQSSIAMKNDEMGTELIDQLT 780
                   ************************************************************

SMC3_A,g38923      SEERDLLSRLNPEITDLKERFLMCKNSRIEIETRKEELETNLSTNLIRRQKELEAIISSA 840
SMC3_B,g36126      SEERDLLSRLNPEITDLKERFLMCKNSRIEIETRKEELETNLSTNLIRRQKELEAIISSA 840
                   ************************************************************

SMC3_A,g38923      DSRTLPLEAEAKEQELKSSKRNLDELTSLLKANVDAINNFTRKMDDLKRKRDDLKTREAI 900
SMC3_B,g36126      DSRTLPLEAEAKEQELKSSKRNLDELTSLLKANVDAINNF-RKMDDLKRKRDDLKTREAI 899
                   **************************************** *******************

SMC3_A,g38923      LEQSVQDGAKDLEQLMNSRSTYLAKQEECTKKIRDLGSLPADAFEAYKRKNKKQLHKMLY 960
SMC3_B,g36126      LEQTVQDGAKDLEQLMNSRSTYLAKQEECTKKIRDLGSLPADAFEAYKRKNKKQLHKMLY 959
                   ***:********************************************************

SMC3_A,g38923      DCNEQLKKFSHVNQKALDQYVNFTEQREQLQRRRAELDAGDVKIKELISVLDQRKDESIE 1020
SMC3_B,g36126      DCNEQLKKFSHVNQKALDQYVNFTEQREQLQRRRAELDAGDVKIKELISVLDQRKDESIE 1019
                   ************************************************************
```

```
SMC3_A,g38923       RTFKGVARHFREVFSELVQGGHGYLVMMKKKDGDAVDDD--DEDEDGPRDPGPEGRIEKY 1078
SMC3_B,g36126       RTFKGVARHFRKVFSELVQGGHGYLVMMKKKDGNAVDDDDDDEDEDGPRDPGPEGRIEKY 1079
                    ***********:*********************:*****  *******************

SMC3_A,g38923       IGVKVKVSFTGKGETQSMKQLSGGQKTVVALTLIFAIQRCDPAPFYLFDEIDAALDPQYR 1138
SMC3_B,g36126       IGVKVKVSFTGKGETQSMKQLSGGQKTVVALTLIFAIQRCDPAPFYLFDEIDAALDPQYR 1139
                    ************************************************************

SMC3_A,g38923       TAVGNMIRRLADMADTQFIATTFRPEIAKVADKIYGVTHKNRVSYINVVSKEQALDFIEH 1198
SMC3_B,g36126       TAVGNMIRRLADMADTQFIATTFRPEIAKVADKIYGVTHKNRVSYINVVSKEQALDFIEH 1199
                    ************************************************************

SMC3_A,g38923       DQTHNAS*      1205
SMC3_B,g36126       DQTHNAS*      1206

                    ********
```

ESD4 – 85.44 % identity

```
ESD4_B,g25900       ------------------------------------------------------------ 0
ESD4_A,g3140        MLVAMSLHARRPEAALRRCRWPCPSTLSLHRWVGRPRAHTGGRARPWSLHPTFAGGHGSS 60


ESD4_B,g25900       ----------MDNEAIQQQQSNVGSPAGAGRGGHMLADLCPWEVTVQHSDERDGVEDAEA 50
ESD4_A,g3140        PSRHALQQLTMDNEATQQQQSNVGSPAGAGRGGHVLADFCPPEVTVQHGDERDGIEDAEA 120
                              ***** ******************:***:** ******.*****:*****

ESD4_B,g25900       TPPLALGPDEDLPSSCRKNLSKAKRRKKSHNSNRTTNILSQRTSSFLHEKYDGDQEEILN 110
ESD4_A,g3140        TLSLALGPDEDLPSSGRKNLSKVKRRKKSHNSNRTTNILSQ---SFLQQKCDGDQEEMLN 177
                    *  ************ ******.******************   ***::* ******:**

ESD4_B,g25900       NTKKDNGLPLCESEDTSLDSRSKISMSIVAIPEDYVSNQECDGDQEEMLNNAKKDNTLPL 170
ESD4_A,g3140        NTKKDNGLSFCESEETSLDSRSAISVSIVAIPQDYVCNQECDGDQEELLNNAKKDNALPL 237
                    ******** :****:******* **:******:***.**********:********:***

ESD4_B,g25900       CEYEET-SLDNKSEKSVSIVAIPEDYVCNQECDGDREEMLNDIEKDNGLSLRESEETSLD 229
ESD4_A,g3140        CESEETSSLDNKSKKPVSIVAIPEDYVCNQECDGDQQEMLNDTEKDNGLPLCESEETSLD 297
                    ** *** ******:* *******************::***** ****** * ********

ESD4_B,g25900       SKYEKSVSIVAIPEDYVCNQADLDIIEPIKKFPYKPGKEQVVLIDDAFIDRMNMECLFQP 289
ESD4_A,g3140        SKSEKLVSILAIPEDYVCNQADLDIIEPIKKSLINRERNKLCS--SMMLS*--------- 345
                    ** ** ***:*********************   :  ::::    . ::.

ESD4_B,g25900       NAFLNDQVINAYITLLRAQDHLKLRACGKVFLENSLISSILRRDGDDKIKMEDLYPTGDK 349
ESD4_A,g3140        ------------------------------------------------------------ 345


ESD4_B,g25900       NGISTVKKRVLSYLDHDMVFIPINIENTHWYLGVVNAKEREIQVLDSMGTIFGRQDLILT 409
ESD4_A,g3140        ------------------------------------------------------------ 345


ESD4_B,g25900       IKGLQKQIDIVSQHKNLNGHKWPDIQVSSWPVREIHFEQKMQTDGCSCGLFLLKYVEHWT 469
ESD4_A,g3140        ------------------------------------------------------------ 345


ESD4_B,g25900       GEGLSKNITQEDMTQFRTKLAAILLSSDLNKRKGSLLVKVDDEAIGSQSEVEILQSSNSP 529
ESD4_A,g3140        ------------------------------------------------------------ 345


ESD4_B,g25900       CKRKNYHQTPASCSPDRTMDPFLSCGLSTFDMPVTKEDMIDLLCDYLMTIDDAETLETSW 589
ESD4_A,g3140        ------------------------------------------------------------ 345


ESD4_B,g25900       VRSFQPYNITLTVRQLQASLRMNQPMPTDCFNMGVRLLAYRENKRLTSANLMISKHYMDL 649
ESD4_A,g3140        ------------------------------------------------------------ 345


ESD4_B,g25900       RFSTTYEAAQKPRSKKLNVQELAKSLETWPYMKYDASLCRFLLMPWKRGGLNLFVFDREE 709
```

```
ESD4_A,g3140       ------------------------------------------------------------ 345


ESD4_B,g25900      KTLTVLDPTPIPDWCKDMPYKNYVRRIINVSNGYLLAMGVQAPERAVDVFAWKHILPSGI 769
ESD4_A,g3140       ------------------------------------------------------------ 345


ESD4_B,g25900      PVIEDRNLNAFILLQFMSAWNNGKLMPISMDLKRLRKKFVIDLLAYDGNSRRCMIPLSIR 829
ESD4_A,g3140       ------------------------------------------------------------ 345


ESD4_B,g25900      EYLSRITGIRQ   840
ESD4_A,g3140       -----------   345
```

ESD4 – 40.82 % identity

```
ESD4_B,g37251      -----MVETRRQKRVNPQDNEDEQVPNFSMPASPAAAEHHIAVEVCMEAPPSRRTRARCA 55
ESD4_A,g56148      MNIAAIIGHTKFEEFRPTSSANEHIHALGVSYEPRSLEQSI------------------- 41
                        ::   : :...* .. :*::  :.:  .* : *: *

ESD4_B,g37251      ASQEAPPSHRTRARCAASRATTTASDSDAPPSSSSTADFWK-QANDALKPRPMKGAVWTD 114
ESD4_A,g56148      -------------------------------NKQQMEQIFQRMNNNHLVFQNDMGRQFMN 70
                                                  ....  ::::   *: *  :   *  : :

ESD4_B,g37251      EE------NILFCNCCVAMIEAGEMGKREPSLKGDMLEKFCERFNSSSEERQRREPKQFL 168
ESD4_A,g56148      LEHKLDAKLLAYGEKIAEIRMGGDVGFRISKLEDDFAE--------------------LK 110
                    *       : : :  . :  .*::* *  .*:.*: *                    :

ESD4_B,g37251      KKFEKLKELYDK---------YH-EHSALGNER-------------RL-----RMKTKDA 200
ESD4_A,g56148      REFQELRQLLLAHLQSMTTAPYIQERSTTQPEKTTEFFKPTEASTRQDKIMAENMVPQDA 170
                   ::*::*::*             *  *:*:   *:             :      .*  :**

ESD4_B,g37251      TKDLKDLVHFPPKYYDLLHKIFEKPENLNPINNIQDRDDWFEKYKNQSTKYLDYFTLTPE 260
ESD4_A,g56148      P--TTQVTPQKPAFQDKIMAENTVPED-----------APTHQIAPLKPAFDNDYLITTE 217
                       .::.   * : * :      **:              .:    .  : : : :* *

ESD4_B,g37251      DCEAIKFIQDSYQYAEVVDIEGNLLRVLQLRPFVYGCCVKDDLINAYAHIAASEEKNNNK 320
ESD4_A,g56148      DAEAFYFITHSYAEAEVVQINDLVLRIEQLRTHLTGGFIHDQAINAYAHISSVET--DST 275
                   *.**: ** .**  ****:*:. :**: *** .: *  ::*: *******:: *   :..

ESD4_B,g37251      GFITTFEAQKLSQDNGELDD--KRRSWVINVGKKCLEKELIFIPVFNKQKYDWSLLVLNK 378
ESD4_A,g56148      SFIPTFQVQKLLGETGGINNPKQTKKWAELIAKKCIGKNLVFVPMN-VNTNHWVLLVLNF 334
                   .** **:.***  :.* :::  : :.*.  :.***: *:*:*:*:   :. .* *****

ESD4_B,g37251      KEEGGEFQILSP---LPGLRNETVEKTLVKSLQKCIDEAVKDGQST----VDLSQWEIKD 431
ESD4_A,g56148      I--KGEVQILNSLASNPNNRDVVKEHTVVGNIQECIDSSIADGSVSVPQPINIMQWETEV 392
                       **.***.     *. *: . *:*:* .:*:***.:: **. :    ::: *** :

ESD4_B,g37251      YSTHIPQQSDMTSSGVYMIKYMLGWDGSKMDQNFTQDDMNVFRLKICCSLLRSKHNKIQR 491
ESD4_A,g56148      YS-NIPQQTDGHSCGAFMLKYMLTWTGDKMSEHFTQAHINIFKRKISSALLRSDCNKLRL 451
                   ** :****:*  *.*.:*:**** * *.**.::*** .:*:*: **..:****. **::

ESD4_B,g37251      ASYDVPIMKKAYLATSQKDDCDD--NDDDDDLQMADNLTDLMTNN*-------------- 534
ESD4_A,g56148      GSYKDLITKAAYDAKRAEIQREEMAQAADGDIQVINNALDASNSNTKTIKRRGRPKKNEA 511
                   .**.  * * ** *.  : : ::  :  *.*:*: :*  *   ..*

ESD4_B,g37251      ------------------------------------------------------------ 534
ESD4_A,g56148      AENDGKDISNPIDASNSNKRKRGRPKKNDKTPQSVKDLLPTTPIANRVERPNRRVSNPGP 571


ESD4_B,g37251      ----------    534
ESD4_A,g56148      LQLSPYSKF*    580
```

ULP1B – 85.44% identity

```
ULP1B_B,g40710     ------------------------------------------------------------      0
```

```
ULP1B_A,g3140       MLVAMSLHARRPEAALRRCRWPCPSTLSLHRWVGRPRAHTGGRARPWSLHPTFAGGHGSS      60


ULP1B_B,g40710      ----------MDNEAIQQQQSNVGSPAGAGRGGHMLADLCPWEVTVQHSDERDGVEDAEA      50
ULP1B_A,g3140       PSRHALQQLTMDNEATQQQQSNVGSPAGAGRGGHVLADFCPPEVTVQHGDERDGIEDAEA      120
                              ***** ******************:***:** ******.*****:*****

ULP1B_B,g40710      TPPLALGPDEDLPSSCRKNLSKAKRRKKSHNSNRTTNILSQRTSSFLHEKYDGDQEEILN      110
ULP1B_A,g3140       TLSLALGPDEDLPSSGRKNLSKVKRRKKSHNSNRTTNILSQ---SFLQQKCDGDQEEMLN      177
                    *  ************ ******.******************   ***::* ******:**

ULP1B_B,g40710      NTKKDNGLPLCESEDTSLDSRSKISMSIVAIPEDYVSNQECDGDQEEMLNNAKKDNTLPL      170
ULP1B_A,g3140       NTKKDNGLSFCESEETSLDSRSAISVSIVAIPQDYVCNQECDGDQEELLNNAKKDNALPL      237
                    ******** :****:******* **:******:***.**********:********:***

ULP1B_B,g40710      CEYEET-SLDNKSEKSVSIVAIPEDYVCNQECDGDREEMLNDIEKDNGLSLRESEETSLD      229
ULP1B_A,g3140       CESEETSSLDNKSKKPVSIVAIPEDYVCNQECDGDQQEMLNDTEKDNGLPLCESEETSLD      297
                    ** *** ******:* *******************::***** ****** * ********

ULP1B_B,g40710      SKYEKSVSIVAIPEDYVCNQADLDIIEPIKKFPYKPGKEQVVLIDDAFIDRMNMECLFQP      289
ULP1B_A,g3140       SKSEKLVSILAIPEDYVCNQADLDIIEPIKKSLINRERNKLC--SSMMLS*---------      345
                    ** ** ***:*********************   :  ::::   .. ::.

ULP1B_B,g40710      NAFLNDQVINAYITLLRAQDHLKLRACGKVFLENSLISSILRRDGDDKIKMEDLYPTGDK      349
ULP1B_A,g3140       ------------------------------------------------------------      345


ULP1B_B,g40710      NGISTVKKRVLSYLDHDMVFIPINIENTHWYLGVVNAKEREIQVLDSMGTIFGRQDLILT      409
ULP1B_A,g3140       ------------------------------------------------------------      345


ULP1B_B,g40710      IKGLQKQIDIVSQHKNLNGHKWPDIQVSSWPVREIHFEQKMQTDGCSCGLFLLKYVEHWT      469
ULP1B_A,g3140       ------------------------------------------------------------      345


ULP1B_B,g40710      GEGLSKNITQEDMTQFRTKLAAILLSSDLNKRKGSLLVKVDDEAIGSQSEVEILQSSNSP      529
ULP1B_A,g3140       ------------------------------------------------------------      345


ULP1B_B,g40710      CKRKNYHQTPASCSPDRTMDPFLSCGLSTFDMPVTKEDMIDLLCDYLMTIDDAETLETSW      589
ULP1B_A,g3140       ------------------------------------------------------------      345


ULP1B_B,g40710      VRSFQPYNITLTVRQLQASLRMNQPMPTDCFNMGVRLLAYRENKRLTSANLMISKHYMDL      649
ULP1B_A,g3140       ------------------------------------------------------------      345


ULP1B_B,g40710      RFSTTYEAAQKPRSKKLNVQELAKSLETWPYMKYDASLCRFLLMPWKRGGLNLFVFDREE      709
ULP1B_A,g3140       ------------------------------------------------------------      345


ULP1B_B,g40710      KTLTVLDPTPIPDWCKDMPYKNYVRRIINVSNGYLLAMGVQAPERAVDVFAWKHILPSGI      769
ULP1B_A,g3140       ------------------------------------------------------------      345


ULP1B_B,g40710      PVIEDRNLNAFILLQFMSAWNNGKLMPISMDLKRLRKKFVIDLLAYDGNSRRCMIPLSIR      829
ULP1B_A,g3140       ------------------------------------------------------------      345


ULP1B_B,g40710      EYLSRITGIRQ*   840
ULP1B_A,g3140       ------------   345
```

NCAPH – 94.96 % identity

```
css2_B,g47463       MPPAEDAPPQTPPPARGTAAALRVLLQSPPPAFPLGSNDDQQERARARAAARAASVRRRS      60
css2_A,g47542       MPPAEDAPPLTPPPARGTAAASRVLLQSPPPAFPLGSNDDQQERARARAAARAASVRRRS      60
```

```
                        ********* *********** **************************************

css2_B,g47463      LAASIAPSKDPRHDLLNREQVMDLFHNCIKLASENKINQKNTWELGLIDHLSEIIQAGAD      120
css2_A,g47542      LAASIAPSKDPRHDLLNREQVMDLFHNCIKLASENKINQKNTWELGLIDHLSEIIQAGAD      120
                   ************************************************************

css2_B,g47463      EDEETNFQKASCTLEAGVKIYSLRVDSVHSEAYKVLGGINRAGRGEEADLEEGSNVEPAQ      180
css2_A,g47542      EDEETNFQKASCTLEAGVKIYSLRVDSVHSEAYKVLGGINRAGRGEEADLEEGSNVEPAQ      180
                   ************************************************************

css2_B,g47463      DEGINKKNADRRISPASTLESSFEALNVKKFDVAFTVDPLYHQTTAQFDEGGAKGLLLYN      240
css2_A,g47542      DEGINKKDADRRISPASTLESSFEALNVKKFDVAFTVDPLYHQTTAQFDEGGAKGLLLYN      240
                   *******:****************************************************

css2_B,g47463      LGVYGSCHVLFDSFEAPDNCILSDMQTEQAEAIDLSFAKEQIEEMVTQIHLCDDISPTLR      300
css2_A,g47542      LGVYGSCCVLFDSFEAPDNCILSDMQTEQAEAIDLSFAKEQIEEMVTQMRLCNDISPTLR      300
                   ******* ****************************************::**:*******

css2_B,g47463      DIVAQFDEENQRPSHRLSPGQMPVMEDPMDEDNEADDDDSMLPDSGTWDFGGCHDHEDAY      360
css2_A,g47542      DIVAQFDEENQRPSHRLSPGQMPVMEDPMDEDNEADDNDSMQPDSGTWDFGGCHDHEDAY      360
                   *************************************:*** ******************

css2_B,g47463      NENCNPMDSISTNYQEEFNEYTVEIPQGTIVDERLEKIADLLLLGMGSSKANAWAGPEHW      420
css2_A,g47542      NENCNPMDSISTNYQEEFNEYTVEIPQGTIVDERLEKIADLLLLGMGSSKANAWAGPEHW      420
                   ************************************************************

css2_B,g47463      KYRKAKDLEAVPTSSGDSEITNKTQKRSKDKPDIDFTKALDNEHPNIFAAPKNPKLLVLP      480
css2_A,g47542      KYRKAKDLEAVPTSSGDSEITNKMKKRSKDKPDIDFTKALDNEHPNIFAAPKNAKSLLLP      480
                   *********************** :**************************** * *:**

css2_B,g47463      ANRAMCSNKLPEDCHYQPESLVKLFLLPDVLCLAKRRRKSLDAPVDNGDEFIPSEPWEDD      540
css2_A,g47542      ANRAICSNKLPEDCHYRPESLVKLFLLPDVLCLAKRRRQSLDAPLDNGDEFIPSEPWEDG      540
                   ****:***********:*********************:*****:**************.

css2_B,g47463      SFCTDHVDEGHVCSDLEEPVNLINKPRQVNKIDIQYDKVSKQVDVHALKEVLWNHIHASA      600
css2_A,g47542      SFCTDHVDEGHMCSDVEEPINLINKPRQVNKIDIQYDKVSKQVDVHALKEVLWNHIHASA      600
                   ***********:***:***:****************************************

css2_B,g47463      ETDGQEREETGSPLCLSRVLH---------VTSDISPHLYFICLLHLANEHSLKLCDRPT      651
css2_A,g47542      ETDGQEREETGSPLCLSQVLHDLPSSNPDAVTPDISPHLYFICLLHLANEHSLKLCDRPT      660
                   *****************:***         ** ***************************

css2_B,g47463      LDEIDIYMPTSPLVK*       666
css2_A,g47542      LDEIDIYMPTSPPVK*       675
                   ************ ***
```

NCAPG – 98.2% identity

```
css3_B,g10797     MAPAVAVTGAGDSDRLAREVARVLDECYASHAVHPRKLRELAALRSSSSGG-GGGGGPFL  59
css3_A,g5388      MAPAAAVTGAGDSDRLAREVARVLDECYASHAVHPRKLRELAALRSSSSGGGGGGGGPFL  60
                  ****.********************************************** ********

css3_B,g10797     AAFCVAVTPLFALARRSAGSDRIARFVAAFASASAS-SADGGGSGNGFLEEFLRFLVTAS  118
css3_A,g5388      AAFCVAVTPLFALARRSAGSDRIARFVAAFASASASSSADGGGSGNGFLEEFLRFLVTAS  120
                  ************************************ ***********************

css3_B,g10797     KAAHRPARFRACQIISEIIMRLPDDAEVSDQIWDEAIDAMKVRVQDKIAAIRTFAVRALS  178
css3_A,g5388      KAAHRPARFRACQIISEIIMQLPDDAEVSDEIWDEVIDAMKVRVQDKIAAIRTFAVRALS  180
                  ********************:*********:****.************************

css3_B,g10797     RFAIDGEDGGIVDLFLGTLDIEQNAEVRKAIVFSLPPSNNTLESVVESTLDISESVRRAA  238
css3_A,g5388      RFAIDGEDGGIVDLFLRTLDIEQNAEVRKAIVFSLPPSNNTLESVVESTLDISESVRRAA  240
                  **************** *******************************************

css3_B,g10797     YSVLSTKFPLQSLTIKQRTTVLHRGLSDRSVSVNNVCLKMLKDEWLVKNCEGDVISLLRF  298
css3_A,g5388      YSVLSTKFPLQSLTIKQRTTVLHRGLSDRSVSVNNVCLKMLKDEWLVKNCGGDVISLLRF  300
                  ************************************************** *********
```

```
css3_B,g10797     LDVETYESVGESVMAVLLKDGALRVHDGHSIRQYITANGEKEQDSNIQLMDAEVALYWRI  358
css3_A,g5388      LDVETYESVGESVMAVLLKDGALRVHDGHSIRQYITANGEKEQDSNIQLMDAEVALYWRI  360
                  ************************************************************

css3_B,g10797     MCKHLQAEAQAKGSEAAATTGAEAAVYASEATDKNDLLDNVLPSTITDYVDLVKAHLSAG  418
css3_A,g5388      MCKHLQAEAQAKGSEAAATTGAEAAVYASEATDKNDLLDNVLPSTITDYVDLVKAHLSAG  420
                  ************************************************************

css3_B,g10797     PNYHFTSRQLLLLGEMLDFSDTMNRKIASSFLHELLIRPLEHEVDDDGNQIAIGDGVSLG  478
css3_A,g5388      PNYHFTSRQLLLLGEMLDFSDTMNRKIASSFLHELLIRPLEHEVDDDGNQIAIGDGVSLG  480
                  ************************************************************

css3_B,g10797     GDKDWAKAVAELAKKVHSSVGEFEMVVSSVVEELARPCRERTADFMQWIHCLAVTGLLLQ  538
css3_A,g5388      GDKDWAKAVAELAKKVHSSVGEFEMVVSSVVEELARPCRERTADFMQWMHCLAVTGLLLQ  540
                  ************************************************:***********

css3_B,g10797     NTSTLRNLQATAIEPSELLHSLLLPAAKQNHVDVQRAALRCLCLLGLLENRPNAELVKQL  598
css3_A,g5388      NTSTLRNLQATAIEPSELLHSLLLPAAKQNHVDVQRAALRCLCLLGLLENRPNAELVKQL  600
                  ************************************************************

css3_B,g10797     RLSFINGPDLVSAIACKALIDLVTWHGPQEIDRAIGIDLPDPSYKKSQFTQVDLSDMNDD  658
css3_A,g5388      RLSFINGPDLVSAIACKALIDLVTWHGPQEIDRAIGIDSPDPSYKKSQFTQVDLSDMNDD  660
                  ************************************** *********************

css3_B,g10797     DLNIGVLDILFSGFYKGDWEFDLEGDNHDKIPTILGEGFAKILLLSGNFASIPIDLHTVI  718
css3_A,g5388      DLNIGVLDILFSGFYKGGWEFDLEGDNHDNIPTILGEGFAKILLLSGNFASIPIDLHTVI  720
                  *****************.***********:******************************

css3_B,g10797     VAQLIRLYFSEETKELERLKQCLSVFFQHYPALSDKHKSCISNAFVPVMKAMWPGLYGNA  778
css3_A,g5388      VAQLIRLYFSEETKELERLKQCLSVFFQHYPALSDKHKSCISNAFVPVMKAMWPGLYGHA  780
                  **********************************************************:*

css3_B,g10797     GGSPVVISKRRKLAVQASRFMVQMVQTQLLSTESMGQASKSPESAPVSANVSNNFDISEE  838
css3_A,g5388      GGSPVVISKRRKLAVQASRFMVQMVQTQLLSTESMGQASKSPESAPVSANVSNNFDISEE  840
                  ************************************************************

css3_B,g10797     GLAIRIALEVAGCPDKKTAAGKAYALALCKVAVLLRFRQSEQKAIKCMRGLVNHLAASVA  898
css3_A,g5388      GLAIRIALEVAGCPDKKTAAGKAYALALCKVAVLLRFRQSEQKAIKCMRGLVNHLAASVA  900
                  ************************************************************

css3_B,g10797     SDKELVKELAQMAARLKALDACPDEELSQDDADVIFNKLGLDDGFKLNSNQAVPPTPAPR  958
css3_A,g5388      SDKELVKELAQMAARLKALDACPDKELSQDDADVIFKKLGLDDGFKLDSNQAVPPTPAPR  960
                  ************************:***********:**********:************

css3_B,g10797     SARPPAPARRRARQAPPPSSSDESDEGGDVSVPESVSRVPATPSMTAAAHSQRASKTTAL  1018
css3_A,g5388      SARPPAPARRRARQAPPPSSSDESDEGGDVSVPESVSRIPATPSMTAAARSQRASKTAAL  1020
                  **************************************:**********:*******:**

css3_B,g10797     SKMSAKPPAIASDGSESDDQSDVTSEEDSSAEESS*      1053
css3_A,g5388      SKMSAKPPAIASDGSESDDQSDVTSEEDSSAEESS*      1055
                  ************************************
```

HGV2 – 93.29 % identity

```
HGV2_B,g43483      MASSSENTGAPPEVEQQPQAPPTPNPEPTEAAAEEEEEEEPRTLERAQELFDRGAKAIED 60
HGV2_A,g31157      MASSSENTGAPPEVEQQPQAPPTPNPEPTEAAAEE--EEEPRTLERAQELFDRGAKAIED 58
                   ***********************************  ***********************

HGV2_B,g43483      EDFVEAVDCLSQALEIRTSHYGELAPECASTYFKYGCALLYKAQEESDFLGNVPKSVPNE 120
HGV2_A,g31157      EDFVEAVDCLSQALEIRTSHYGELAPECASTYFKYGCALLYKAQEESDFLGNVPKSVPNE 118
                   ************************************************************

HGV2_B,g43483      ESVKSTASKDDSGTSKVSCTNVEDAMSSKKADAEEGQNSNGKDQETGNGEVEKDDEDDDN 180
HGV2_A,g31157      ESVKSTTSKDDSGTSKVSCTYVAYLLQFKDFLTIETSISLNVSL*--------------- 162
                   ******:************* *   :. *.  : * . * . .
```

```
HGV2_B,g43483      DEKMGDEEDNDLDLSWKMLDIARAIVEKTPDNSMEKVKIYSALAEVATEREDIDNSLSDY 240
HGV2_A,g31157      ------------------------------------------------------------ 162


HGV2_B,g43483      MKALSMLEHLVEPDHRRVVELNFRICLVYELVSKIGDAIPYCAKAISLCKSRIQSLKSSK 300
HGV2_A,g31157      ------------------------------------------------------------ 162


HGV2_B,g43483      DALLAGKDGDASAAEAEGGSEKSDAEKELEQLTSILPDLEKKLEDLEQANPSPAMDEMLK 360
HGV2_A,g31157      ------------------------------------------------------------ 162


HGV2_B,g43483      TIASRVTDAMPRAASFTSSQMATSSNGFDSSVLSTAATTGSTGSTVTDLGVVGRGVKRAS 420
HGV2_A,g31157      ------------------------------------------------------------ 162


HGV2_B,g43483      IKPISAEPAAKKPALDSPSVQGDSSINSEVVPTTQNGDESVSK*    463
HGV2_A,g31157      --------------------------------------------    162
```

ANAPC15 – 98.97% identity

```
ANAPC15_B,g3841      MLQFPALMRQWPSPPLLPASTLLPVPATSQEDELLLAMAESDLDDKLNEIRKTNSHLVII      60
ANAPC15_A,g6253      MLQFPALMRQWPSPPLIPASTLLPVPATSQEDELLLAMAESDLDDKLNEIRKTNSHLVII      60
                     ****************:*******************************************

ANAPC15_B,g3841     *      96
ANAPC15_A,g6253      GKPTGDTKEEYDAEVEDDDADNVEESDGDDFDQETG*  96
                     *************************************
```

CYCB1_5 – 66% identity

```
g5077.t1       MATRNHHA----ASAAQPANRGAARIAGKQNGA-ATSRPDAARRVLGDVGNVVSDVLNGK     55
g13110.t1      MATRNHRAAVPAAAAPQPVNRGAARIAGKQKDAAAAGRPNATRAALGDIGNVAPSDVLDG     60
               ******:*    *:* **.***********:.* *:.**:*:* .***:***. . : .

g5077.t1       NTLPEGIHGPITMSFGAALVNDVLANNNTIAPAQPVAARTITNPATIVPAKNTNTPHGEK     115
g13110.t1      IKLPEGIHRPITRSFGAQLLKQQALAKNAGAPAQPVAARAVTKPVKKVPAKNIPRPEQE-     119
                .****** *** **** *:::    :*: *********::*:*.. *****   *. *

g5077.t1       APKVNRKPSDGVAGSSSS------CSVQRNMRTKLVRTPSTILSDLFEAACGLTEKPKEL     169
g13110.t1      ----NRKPSEGAAKDSKGNMNTSEGVAAVQRRKKLVCTLSTVLSARSKAACGLTEKPKPL     175
                   *****:*.* .*..        .  : *.*** * **:**   :********** *

g5077.t1       IEDIDKFDGDDQFAVVDYVEDIYKFYMTAEHESRPNDYMGNQPEITSKTRASLVDRLIHS     229
g13110.t1      VEDIDKFDGDNQLALVDYVEEIYTFYKTAQHEIRPIDYMGNQPEINLNMRATLTDWLIDS     235
               :*********:*:*:*****:**.** **:** ** *********. : **:*.* **.*

g5077.t1       HQRFHLKPETLYLTIYIVDQYLSLQPVPSMELGLVGAAAMLIAWKYEEAWAEKEKIFIEI     289
g13110.t1      HLRFHLMPETLYLTIYIVDRYLSLQPVPRREFQLVGMAAMLIACKYEEIWAPEVNDFIEI     295
               * **** ************:********  *: *** ****** **** ** : : ****

g5077.t1       --RPFDRHQILHMEKAILNSMNWELAVPTPYHFLLRFAKAASSDDEQLQHMVHFFGELAL     347
g13110.t1      AARAFSRTQILVTEKAILNSIEWNLTVPTPYHFLLRFAKAAGSADEQLQHMIYFFGELAL     355
                 * *.* ***  *******::*:*:***************.* *******::*******

g5077.t1       MDYGMMMTYASRVAACAVYAARLTLKKIPFWTQTLKHHTGLEEQQLIEGARILVSSHAAA     407
g13110.t1      MEYGMVTTYPSTIAACAVYAARLTLRKSPLWTETLRHHTGLHEPQLMEGARMLLRSHA-A     414
               *:***: ** * :************:* *:**:**:*****.* **:****:*: *** *

g5077.t1       PDAKLKAVYQKYAVEQFGKVALHPPAALSDLV*      439
g13110.t1      PDAKRKTVHEKYATEQFGRVALHPPAALPDLV*      446
               **** *:*::***.****:********* ****
```

putative helicase – 53,07% identity

```
g5838.t1        MQMNVTPLSNIRPGILDYTICVRVSRLWEFRGDSEENDVKHLDLVLIDQKANAIYAEIPP    60
g31445.t1       ------------------------------------------MVVIDQKGYAMYCEVPP    17
                                                          :*:****. *:*.*:**

g5838.t1        EAIPMTKPHLEEGNIVYMMKITVQRSKPEYRVVENPYMIKLNRRTIIIEAKDQPPEFPKY    120
g31445.t1       EVLSHKKQYLEEGKVLYIWKACVERAKPGYRVVDAPYMLKLIMRTEIFEGNSNDTSFPKY    77
                *.:  .* :****:::*: *  *:*:** ****: ***:**  ** *:*.:.:  .****

g5838.t1        TFSLIPFDKLHEFTRKTQKFLDVIGKITAVSNAARVYSSSGDLRIRRVIQLRDLSGNTID    180
g31445.t1       VFSLTPIEILPQYARRTDRFLDVIGKITAISNAAVARNTSGDLMMRRMITLQDHKGNTVD    137
                .*** *:: * :::*:*::*********:**** . .:**** :**:* *:* .***:*

g5838.t1        LSLSGKRALEFDGETVLKVGQQNHVIAIFVGTLMKLYQMKHHFLSGTSACRWYINQNDIP    240
g31445.t1       LSLSGQRALEFDAEAVFDIGQNHHVIAIFVGTLMKIYREDYKFLSGTSACRWYINENDIP    197
                *****:******.*:*:.:**::***********:*: .::************:****

g5838.t1        EIKAFQKSLPAQAEPIQTLYLQSEDDTKQNFEEKTLLQLKEVDPFTEKDKRFQCTVTIAA    300
g31445.t1       AMRTFQRGLPSQVTPVQQLQLQSEDYMEQGVEEKTLFDLKQIDPLNDKNKRFQCTVTLIS    257
                 :::**:.**:*. *:* * *****  :*..*****::**::**:.:*:********: :

g5838.t1        VPERQPWCYRACRSCNCTIRVQRSGFKCSNDNCFTTQFDWKYKIPFIGSDGTYNLEFILF    360
g31445.t1       LAEKEQWCYRACKVCNSTLILGNDGYDCTKDGCSCKQYDWKYKVCFVVADDTYNLQFMFF    317
                : *:: ******: **.*: : ..*:.*::*.*  .*:*****: *: :*.****:*::*

g5838.t1        EKRGAELIGKTAEALRKQYDMLDTPPEILAWIGHKFTFVVKILSTKSINTIDPSFEVVLI    420
g31445.t1       EKKGVELIGKSAETLRKKYDPSSIPPEIAQWIGHKFTFIVKVLFKRSIKSIEPSFEVVMI    377
                **:*.*****:**:***:**   . ****  ********:**:* .:**::*:******:*

g5838.t1        KEKFGKQPTIPTLSPDHGLSMRASSTKFQADEELPLLVPIGSKYIQEQDSSSQLQVHANM    480
g31445.t1       KERHGKQVTLPNITHKD-----------VSNDDLPPLVTISSKKKIKQASLSSTPQFTDI    426
                **:.*** *:*.:: ..           ::::** ** *.**   :* * *.   .:::

g5838.t1        HTMDIDSS--SYDSNKRALDLTDDENQDDSLLQPKNKRP-R*-- 518
g31445.t1       QHMDIDQQSDAWDTTEKHIQSQIDDEDDERHEEQKRKKLKINE* 469
                : ****..  ::*:.:: ::   *:::*:   : *.*:
```

TPR repeat-containing protein ZIP4-like – 97% identity

```
g3867.t1        MKISELSPEYRISQLSPECRSPPAHAALLTDLNRVVTDVEALDASDSSSLEKLAADLRCV    60
g10502.t1       MKISELSPEYRISQLSPECRSPPAHAALLTDLNRVVTDVEALDASDSSSLEKLAADLRCV    60
                ************************************************************

g3867.t1        LTNLASAVSSSSSGLNGAFRLKVWNLAFRLWNACVDRANHKFPARGPEAAVAETEIRQAA    120
g10502.t1       LTNLASAASSSSSGLNGAFRLKVWNLAFRLWNACVDRANHKFPARGPEAAVAETEIRQAA    120
                *******.****************************************************

g3867.t1        PELLLIAGLPEGVPNAAAKAASLFHRTGLVWLDLGRADLASACFEKATPLVCAADT--GR    178
g10502.t1       PELLLIAGLPEGVPNAAAKAASLFHRTGLVWLDLSRADLASACFEKATPLVCAADTEEDR    180
                **********************************.*********************  .*

g3867.t1        DILLDLNLARARTASSQGKHALAVALLSRSKPLAAASSQGFKALAEPYLLLGKAALATRS    238
g10502.t1       DILLDLNLARARTASSQGKHALAVGLLSRSKPLAAASSEGFKALAEAYLLLGKAALATKS    240
                ************************.*************:******* ***********:*

g3867.t1        PDPAIDASSLLTEALDLCEKAAASPCCATPTTPRSTPATTKLQVIKDQCLRFLAAERLEA    298
g10502.t1       PDPAIDASSLLTEALDLCEKAAASPCCATPTTPRSTPATTKLQLIKDQCLRFLAAERLEA    300
                *******************************************:****************

g3867.t1        NDYEGTLQCTRASRASPGLGKEHSSIAFMALRACLSSGKLVDAKRELGRLMANQEAPEFL    358
g10502.t1       NDYEGTLQCTRASRASPGLGKEHSSIAFMALRACLSSGKLVDAERELGRLMANEEAPEFL    360
                *******************************************:*********:******

g3867.t1        CVSAAELYLASAGLDAALKVLVALAARCRASAAAAAVRVLKTVVQGAGGGAGLARAIAEL    418
g10502.t1       CVSAAELYLASAGLDAALKVLVALASRCRASAAAAAVRVLKTVVQGAGGGAGRARAIAEL    420
                *************************:************************** *******
```

```
g3867.t1        VSDERVVALFNGTANTHERDTMHALLWTCGSEHFHAKNCEIAADLIERSMLYVSRDEESR     478
g10502.t1       VSDERVVALFNGPANTHERDTMHALLWTCGSEHFHAKNCEIAADLIERSMLYVSRDEESR     480
                ************ ***********************************************

g3867.t1        SRRAKCFRVLCLCHMALRHLDRAQEFITEAEKVEPNIHCAFLKFKILLHKKEDDEAIKLM     538
g10502.t1       SRRAKCFRVLCLCHMALRHLDRAQEFITEAEKVEPNIHCAFLKFKILLHKKDDDEAIKLM     540
                ***************************************************:********

g3867.t1        KTMVGYVDFNPHFLALSIHEAIGCKSFRVAVASLTFFLGLYSVGKPMPMGEAAVHRNLIA     598
g10502.t1       KTMVGYVDFNPHFLALSIHEAIGCKSFRVAVASLTFFLGLYSVGKPMPMSEAAVHRNLIA     600
                *************************************************.**********

g3867.t1        LLLLEPGSEAEILKYSRRAKLLMDELGVETFLGKGTVGLRELNWFAVSSWNMALKVVKEK     658
g10502.t1       LLLREPGSDAEILKYSRRAKLRMDELGVETFLGKGTVGLRELNWFAVSSWNMALKVVKEK     660
                *** ****:************ **************************************

g3867.t1        KYDYSSEFFELAAEFFSSESDLKKGIEMLRRAGKLLPLTSSSAPVTSDPLENNLPFLHTF     718
g10502.t1       KYDYSSEFFELAAEFFSSESDLKKGIEMLRRAGKLLPLTLPSAPVTSDPLENNLPFLHTF     720
                ***************************************  *******************

g3867.t1        NFYQLLNRLDTSAHPQQLQLVKSFAASKAYTPDHLLILGNMASEGTQPNLQVAEFVLKAS     778
g10502.t1       NFYQLLNRLDTSAHPQQLQLVKSFAASKACTPDHLLILGNMASEGTLPNLQVAEFLLKAS     780
                ***************************** **************** ********:****

g3867.t1        ISTALASHSPNYGVISAALRKLVYLSGLQDFSGSMSDAAYDVFQQAYQIVVGLRDGEYPF     838
g10502.t1       ISTALASHSPNYGVISAALRKLVYLSGLQDFSGSMSDAAYDVFQQAYQIVVGLRDGEYPF     840
                ************************************************************

g3867.t1        EEGRWLAITAWNKSYLPGQIGQHSVAKKWMKMGLDLARHFDRMKLYIPGMEECFENFQKL     898
g10502.t1       EEGRWLAITAWNKSYLPGRIGQHSVAKKWMKMGLDLARHFDRMKLYIPGMEECFENFQKL     900
                ******************:*****************************************

g3867.t1        SGKEPYERSQQDGEPSTSMSGTGSMSQPVLV*      929
g10502.t1       SGKEPDECSQQDGEPSTSMSGTGSMSQPVLV*      931
                ***** * ************************
```

protein argonaute MEL1 – 96.38% identity

```
g48565_B       MASRGRG-GGGGQGAGGGRRGEGRGRGVGGRGGYPQPYGRGEHGGGEPGGRGGG------     53
g9296_A        MASRGRGSGGGGQGSGGGRGGEGRGRGVGGRGGYPQPYGRGEHGGGEPGGRGGGMGRGRG     60
               ******* ******:**** **********************************

g48565_B       -----------------MGYQQPPPPPVGNVEGGGGRGRGGVAAAPARPAAPAPRPQAPP     96
g9296_A        IGGRGDGGGRGTGGRGGVGYQQPPPPPLGNVEGGGGRGRGGVAAAPARPAAPAPRPQAPP     120
                                :*********:********************************

g48565_B       VAAPAFPAAASSSAPSPTPAQAPGAPAGAAPVAPLAAGMGRLAVADSNPRPAVPPAPAAV     156
g9296_A        VAAPAFPAAASSSAPSPTPAQAPRAPAGAAPVAALAAGMGRLAVADNNPRPAAPPAPAAV     180
               *********************** ********* ************.*****.*******

g48565_B       RSEAQAAAPARQPPQAPPLSSKGIAPPARPGFGTSGRKVLVRANHFAVQVADNDICHYDV     216
g9296_A        RSEAQAAAPARQPPQAPPLSSKGIAPPARPGLGTSGRKLLVRANHFAVQVADNDICHYDV     240
               *******************************:******:*********************

g48565_B       LINPEPKARRINRVILSELVKVHGATSLARKIPAYDGSKSLYTAGELPFKSMEFVVKLGR     276
g9296_A        LINPEPKARRINRVILSELVKVHGATSLARKIPAYDGSKSLYTAGELPFKSMEFVVKLGR     300
               ************************************************************

g48565_B       REIEYKVTIRYAARPNLFHLKQFLKGQQRDAPYDTIQALDVALRESPSLNYVTLSRSFFS     336
g9296_A        REIEYKVTIRYAARPNLFHLKQFLKGQQRDAPYDTIQALDVALRESPSLNYVTLSRSFFS     360
               ************************************************************

g48565_B       KNFGEVKDIGGGLECWRGYYQSLRPTQMGLSLNIDICSTSFYQSISVVKFVGDCLRLTNP     396
g9296_A        KNFGEGEDIGGGLECWRGYYQSLRPTQMGLSLNIDICSTSFYQSIPVVKFVSDCLRLTNP     420
               ***** :************************************** *****.********

g48565_B       AQPFPNRDRLKLKKALRGVRVETTHQQGKRSIYKITGITPVPLTQLSFSCEEGPQLTVVQ     456
```

```
g9296_A       AQPFSDRDRLKLKKALRGVRVETTHQQGKRSIYKITGITPVPLTQLSFSCEEGPQLTVVQ      480
              **** :******************************************************

g48565_B      YFARRYNYRLHYTAWPCLQSGNDSKPIYLPMEVCQIIEGQRYPRKLSDTQVANILKATCK      516
g9296_A       YFAQRYNYRLRYTSWPCLQSGNDSKPIYLPMEVCQIIEGQRYPRKLSDTQVANILKATCK      540
              ***:******:**:**********************************************

g48565_B      PPQEREDSIIKMVRQNNYSADRMAQVFGITVANQMANVQARVLPAPTLKYHESGKEKTVA      576
g9296_A       RPQEREDSIIKMVRHNNYSADKMAQVFGITVANQMANVQARVLPAPMLKYHESGKEKTVA      600
               *************:******:************************* ************

g48565_B      PSLGQWNMINKKMVNGGTVDSWTCLSFSRIPLHEVNRICEDLAQMCNSIGMRFNPRPVTE      636
g9296_A       PSLGQWNMINKKMVNGGTVDSWTCLSFSRIPLHEVNRICEDLAQMCNSIGMRFNPRPVTE      660
              ************************************************************

g48565_B      VKSASPNHIEGALRDVHTRAPNLQLLIVILPDVSGHYGTIKRICETDIGIVSQCMNPKKN      696
g9296_A       VKSASPNHIEAALRDVHMRAPNLQLLIVILPDVSGHYGTIKRICETDLGIVSQCMNPKKN      720
              **********.****** *****************************:************

g48565_B      KNKQYFENVALKVNVKVGGCNTVLERALVKNGIPFVNDVPTIIFGADVTHPTAGEDSSAS      756
g9296_A       KNKQYFENVALKVNVKVGGRNTVLERALVPNGIPYVTDVPTIIFGADVTHPTAGEDSSAS      780
              ******************* ********* ****:*.***********************

g48565_B      IAAVVASMDWPQVTTYKALVSAQAHREEIIQNLFWTATDPEKGTPVNGGMIRELMISFFR      816
g9296_A       IAAVVASMDWPQVTTYKALVSAQAHREEIIQNLFWTATDPEKGTPVNGGMIRELMTSFFR      840
              ******************************************************* ****

g48565_B      RTARKPRRIIFYRDGVSEGQFSHVLLHEMDAIRKACASMEDGYLPPVTFVVVQKRHHTRL      876
g9296_A       RTGRKPRRIIFYRDGVSEGQFSHVLLHEMDAIRKACASMEDRYQPPVTFVVVQKRHHTRL      900
              **.************************************** * ****************

g48565_B      FPEVHGRRDLTDNSGNILPGTVVDTSICHPSQFDFYLCSHAGIKGTSRPTHYHVLYDEND      936
g9296_A       FPEVHGRRDLTDKSGNILPGTVVDTSICHPSEFDFYLCSHAGIKGTSRPTHYHVLYDENH      960
              ************:******************:***************************.

g48565_B      FSADGLQMLTNSLCYTYARCTRAVSVVPPAYYAHLAAFRARYYDEQGSTDGASVVSGGAA      996
g9296_A       FSADGLQMLTNSLCYTYARCTRAVSVVPPAYYAHLAAFRARYYDEQGSTDGTSVVSGGAA      1020
              ***************************************************:********

g48565_B      AAGGGAPAFRRLPQIKENVKDVMFFC*      1022
g9296_A       AAGGGAPAFRRLPQIKENVKDVMFFC*      1046
              ***************************
```

mediator of RNA polymerase II transcription subunit 23 – 99,75% identity

```
g56330_B      MDGGHGARQPMSPAISASAVLPQQRQMQPHHHPARTAIADLFTLYLGMNSKQRAEDPMRE      60
g44089_A      MDGGHGARQPMSPAISASAVLPQQRQMQPHHHPARTAIADLFTLYLGMNSKQRAEDPMRE      60
              ************************************************************

g56330_B      SPNKLQKRVTALNRDLPPRDEQFISDYEQLRMPFPDAEQLQAVTESVLISFVLQCSSHAP      120
g44089_A      SPNKLQKRVTALNRDLPPRDEQFISDYEQLRMPFPDAEQLQAVTESVLISFVLQCSSHAP      120
              ************************************************************

g56330_B      QSEFLLFATRCLCARGHLRWDSLLPALLNTVSSIEAPMVQGVSVTGAGPATPSSAIAMPN      180
g44089_A      QSEFLLFATRCLCARGHLRWDSLLPALLNTVSSIEAPMVQGVSVTGAGPATPSSAIAMPN      180
              ************************************************************

g56330_B      APNFHPSNPASPLSVMNTIGSPTQSGIDQPVGANVSPIKAAEFSSSAQLGTAARGDQSRR      240
g44089_A      APNFHPSNPASPLSVMNTIGSPTQSGIDQPVGANVSPIKAAEFSSSAQLGTAARGDQSRR      240
              ************************************************************

g56330_B      GAEASYLHHLSCRIILAGLEFNLKPATHAVIFQHMVNWLVNWDQRPHGMDEADVMQTCRL      300
g44089_A      GAEASYLHHLSCRIILAGLEFNLKPATHAVIFQHMVNWLVNWDQRPHGMDEADAMQTCRL      300
              *****************************************************.******

g56330_B      EKPLHEWMHLCLDVIWILVNEDKCRIPFYELVRCNLQFLENIPDDEALVSIIMEIHRRRD      360
g44089_A      EKPLHEWMHLCLDVIWILVNEDKCRIPFYELVRCNLQFLENIPDDEALVSIIMEIHRRRD      360
              ************************************************************
```

```
g56330_B      MVCMHMQMLDQHLHCPTFGTHRFLSQSYPSIAGESVTNLRYSPITYPSVLGEPLHGEDLA      420
g44089_A      MVCMHMQMLDQHLHCPTFGTHRFLSQSYPSIAGESVTNLRYSPITYPSVLGEPLHGEDLA      420
              ************************************************************

g56330_B      NSIPKGGLDWERALRCLRHALRTTPSPDWWRRVLLVAPCYRSQSQQSSTPGAVFSPDMIG      480
g44089_A      NSIPKGGLDWERALRCLRHALRTTPSPDWWRRVLLVAPCYRSQSQQSSTPGAVFSPDMIG      480
              ************************************************************

g56330_B      EAVADRTIELLRLTNSETQSWQDWLLFADIFFFLMKSGCIDFLDFVDKLASRVTSSDQQI      540
g44089_A      EAVADRTIELLRLTNSETQSWQDWLLFADIFFFLMKSGCIDFLDFVDKLASRVTSSDQQI      540
              ************************************************************

g56330_B      LRSNHVTWLLAQIIRIEIVMNTLSSDPRKVETTRKIISFHKEDKSLEANNIGPQSILLDF      600
g44089_A      LRSNHVTWLLAQIIRIEIVMNTLSSDPRKVETTRKIISFHKEDKSLEANNIGPQSILLDF      600
              ************************************************************

g56330_B      ISSSQTLRIWSFNTSIREHLNSDQLQKGKQIDEWWKQMTKASGERMIDFMNLDERETGMY      660
g44089_A      ISSSQTLRIWSFNTSIREHLNSDQLQKGKQIDEWWKQMTKASGERMIDFMNLDERATGMF      660
              ******************************************************* ***:

g56330_B      WVLSFTMAQPACEAVMNWFTSAGMADLIQGPNMQPSERIMMMRETYPLSMSLLSGLSINL      720
g44089_A      WVLSFTMAQPACEAVMNWFTSAGMADLIQGPNMQPSERIMMMRETYPLSMSLLSGLSINL      720
              ************************************************************

g56330_B      CLKLAFQLEETIFLGQAVPSIAMVETYVRLLLIAPHSLFRPHFTTLTQRSPSILSKSGVS      780
g44089_A      CLKLAFQLEETIFLGQAVPSIAMVETYVRLLLIAPHSLFRPHFTTLTQRSPSILSKSGVS      780
              ************************************************************

g56330_B      LLLLEILNYRLLPLYRYHGKSKALMYDVTKIISMIKGKRGEHRLFRLAENLCMNLILSLK      840
g44089_A      LLLLEILNYRLLPLYRYHGKSKALMYDVTKIISMIKGKRGEHRLFRLAENLCMNLILSLK      840
              ************************************************************

g56330_B      DFFFVKKELKGPTEFTETLNRITIISLAITIKTRGIAEVEHMIYLQPLLEQIMATSQHTW      900
g44089_A      DFFFVKKELKGPTEFTETLNRITIISLAITIKTRGIAEVEHMIYLQPLLEQIMATSQHTW      900
              ************************************************************

g56330_B      SEKTLRYFPPLIRDFLMGRMDKRGQAIQAWQQAETTVINQCNQLLSPSAEPNYVMTYLSH      960
g44089_A      SEKTLRYFPPLIRDFLMGRMDKRGQAIQAWQQAETTVINQCNQLLSPSAEPNYVMTYLSH      960
              ************************************************************

g56330_B      SFPQHRQYLCAGAWMLMNGHLEINSANLARVLREFSPEEVTANIYTMVDVLLHHIQFEVQ      1020
g44089_A      SFPQHRQYLCAGAWMLMNGHLEINSANLARVLREFSPEEVTANIYTMVDVLLHHIQFEVQ      1020
              ************************************************************

g56330_B      RGHLAQDLLSKAITNLSFFIWTHELLPLDILLLALIDRDDDPYALRLVISLLEKPELQQR      1080
g44089_A      RGHLAQDLLSKAITNLSFFIWTHELLPLDILLLALIDRDDDPYALRLVISLLEKPELQQR      1080
              ************************************************************

g56330_B      VKNFCNTRSPEHWLKNQHPKRAELQKALGSHLSWKDRYPPFFDDIAARLLPVIPLIIYRL      1140
g44089_A      VKNFCNTRSPEHWLKNQHPKRAELQKALGSHLSWKDRYPPFFDDIAARLLPVIPLIIYRL      1140
              ************************************************************

g56330_B      IENDATDIADRVLAFYSSLLAFHPLRFTFVRDILAYFYGHLPIKLIGRILNLLGVSTKTP      1200
g44089_A      IENDATDIADRVLAFYSSLLAFHPLRFTFVRDILAYFYGHLPIKLIGRILNLLGVSTKTP      1200
              ************************************************************

g56330_B      FSESFAKYLVSSNSSICPPPEYFANLLLNLVNNVIPPLSSKSKSNPADTTRSTFNKHHAS      1260
g44089_A      FSESFAKYLVSSNSSICPPPEYFANLLLNLVNNVIPPLSSKSKSNPADTTRSTFNKHHAS      1260
              ************************************************************

g56330_B      SQPGGIGNTDGQRAFYQNQDPGSYTQLVLETAAIEILSLPVPAAQIVSSLVQIIAHVQAM      1320
g44089_A      SQPGGIGNTDGQRAFYQNQDPGSYTQLVLETAAIEILSLPVPAAQIVSSLVQIIAHVQAM      1320
              ************************************************************

g56330_B      LIQSNSGQGMSGGLGQSSGLPTSPSGAAESSGPNQANSAASGINATNFISRSGYSCQQLS      1380
g44089_A      LIQSNSGQGMSGGLGQSSGLPTSPSGAAESSGPNQANSAASGINATNFISRSGYSCQQLS      1380
              ************************************************************
```

```
g56330_B      VLMIQACGLLLAQLPPEFHMQLYSEAARVIKDCWWLADSSRPVKELDSAVGYALLDPTWA      1440
g44089_A      VLMIQACGLLLAQLPPEFHMQLYSEAARVIKDCWWLADSSRPVKELDSAVGYALLDPTWA      1440
              ************************************************************

g56330_B      SQDNTSTAIGNTVALLHSFFSNLPQEWLESTHTVIKHLRPVNSVAMLRIAFRILGPLLPR      1500
g44089_A      SQDNTSTAIGNTVALLHSFFSNLPQEWLESTHTVIKHLRPVNSVAMLRIVFRILGPLLPR      1500
              *************************************************.**********

g56330_B      LAFARPLFMKTLALLFNVLGDVFGKNPPVPNPNPVEASEIADIIDFLHHAVMYEGQGGPV      1560
g44089_A      LAFARPLFMKTLALLFNVLGDVFGKNPPVPNPNPVEASEIADIIDFLHHAVMYEGQGGPV      1560
              ************************************************************

g56330_B      QSTSKPKLEILTLCGKVIEILRPDVQHLLSHLKIDPTSSIYAATHPKLVQNSS*      1613
g44089_A      QSTSKPKLEILTLCGKVIEILRPDVQHLLSHLKIDPTSSIYAATHPKLVQNSS*      1613
              *****************************************************
```

DUF724 domain-containing protein 7-like – 84,72% identity

```
g3237_B      MAAAASGSAPPPDAAGARRGRGRPRGSKNGSGRGRSRS----------------LRSPSG 44
g5789_A      MAAAASGSAPPPDAAGARRGRGRPRGSKNGSGRGRGRGRSRSLRWSKAPKRPRLSVSPSG 60
             ***********************************.*.                  ****

g3237_B      SPDGPHSPRPAVDHSAPLPPGTEVEVRVDDDGFHGSWFEATVLDFSPARGYCHPARYTVS 104
g5789_A      SPDGPHSPRPAVDLSAPLPPGTEVEVRVDDDGFHGSWFEATVLDFSPARGYRHPARYTVS 120
             ************* ************************************* ********

g3237_B      YVHLLADDDEGVLAEPFAPSHIRPRPPPPAPTDPPRFQTHDIVEAFHNEGWWSGIVVSAP 164
g5789_A      YVHLLADDDEGVLAEPFAPSHIRPRPPPPPPTDPPRFQTHDIVEAFHNEGWWSGIVMSAP 180
             ***************************** **************************:***

g3237_B      DSTDSPDGAGAAGVTVAFPITREVIEFAPGLVRPRRDYVDGEWIPSQVAMIVRPKRAVKV 224
g5789_A      DSTDSPDGAGAAGVTVAFPITREVLEFAPGLVRPRRDYVDGEWIPSQVAMIVRPKRAVKV 240
             ************************:***********************************

g3237_B      YEVGDKVEVVRQRNGYGESWFPATVRVVVDDLSYIVEYFDLEEG-EG-GQKATEYLHWRF 282
g5789_A      YEVGDKVEVVRQRNGYGESWFPATVRVVVDDLSYIVEYFDLEEEGEGGQEKATEYLHWRF 300
             *******************************************  **  :**********

g3237_B      IRPAVEHSPRESEFQLQPGAAVEAYCDGAWSLGVVRTVLGEGEYEIGIVAKKSEMLVTKV 342
g5789_A      IRPAVEHSPRESEFQLQPGAAVEAYCDGAWSPGVVRTVLGEGEYEIGIVAKKSEMLVTKV 360
             ******************************* ****************************

g3237_B      VPLLKPQYKWNGKQWRIATRKRRANTRRRSVSGNSPRSPVEVSSIDQEHSLGKNTLAE-- 400
g5789_A      VPLLKPQYKWNGKQWRIATRKRRANKRRRSVSGNSPRSPVEVSSSDQEHSLGKNTLAEGS 420
             *************************.****************** *************

g3237_B      -------------------------------------------------------GHSGP 405
g5789_A      GHASVSEMDIPLSALCKSPESTRSPNSFVSEKNSPPGSHGIVNSVPMNGLVCASPGHSGP 480
                                                                    *****

g3237_B      VDNQEILSDMVVTDGELNGPVSGRSGDG-------------------------------- 433
g5789_A      VDNQEILSDMVVTDGELNGPVSGRSGDGNDMLSITELRKKMSSARRNSAVNRKQDNLAKS 540
             ****************************

g3237_B      ------ISNIKAGKTHSIQGLEGKIQLKGNMNFSAPDIVLALSVSGTGRTIISPDGLVSI 487
g5789_A      VSVKKCISNIKAGKTHSIQGLEGKIQLKGNMNFSAPDIVLALSVSGTGRTIISPDGLVSI 600
                   ******************************************************

g3237_B      GTERGSSTKVSVGTKRRSSTKVLACKKLANRRGSKELCSPNSSLDVTGTVQQRGRKEVAE 547
g5789_A      GTERGSSTKVSVGTKRGSSTKVLACKKLANRRGSKELCSPNSSLDVTGTVQQRGRKEVAE 660
             **************** *******************************************

g3237_B      PMEECPFALECPNSGTREQLDRTLEDAQNIIELSNPDLFLMVPPGFESMYNGKGINTNDT 607
g5789_A      PMEECPFALECPNSGTREQLDRTLEDAQNIIELSNPDLFPMVPPGFESMDNGKGINTNDA 720
             *************************************** ********* *********:

g3237_B      QFDEEPSGTTNSLIEPKGNGDMCTNHAATKLTESNHVMETAILSLDSPAQQAYGKVDERS 667
g5789_A      QFDEEPSGTTNSLIEPKGNGDMCTNHAATKLTESNHVMETAILSLDSPAQQACGKVDERS 780
```

```
             ***************************************************** *******

g3237_B      VLQNARSSQCIINSSPLRSCPAFESLLPLPQPLSQVSKHQALFVKNSPLWHLVETMHVFK 727
g5789_A      VLQNARSSQCIINSSPLRSCPAFESLLPLPQPLSQVSKHPTLFVKNSPMWHLVETMHVFK 840
             *************************************** :*******:***********

g3237_B      ELPQQPHFLPLQEHLPMLREGMALGMMVSFADLVKITMEASMDNSMEWFKDKIKTISYLE 787
g5789_A      ELPQQPHFLPLQEHPPMLREGMALGMMVSFADLVKITMEASMDNSMEWFEDKIKTISYLE 900
             ************** **********************************:**********

g3237_B      ENGFNVQFIKSNMTELVKVKSELTSYYGLIGKLDSNFVEKTASSSRVGALLDEKDIAAAE 847
g5789_A      ENGFNVQFIKSNMTELVKVKSELTSYYGEIGKLDSKFVEKTASSSRVGALLDEKDIAAAE 960
             **************************** ******:************************

g3237_B      LEQELGRIRQESQRIAKEKEKIDAEVASIKTELSGYKDLCNGAESKAKDILARLRLKRLT 907
g5789_A      LEQELGRIRQESQRIAKEKEKIDAEVASIKTALSGYEDLCNGAESKAKDILARLRLKRLT 1020
             ******************************* ****:***********************

g3237_B      *       907
g5789_A      *       1020
             *
```

histone-lysine N-methyltransferase, H3 lysine-9 specific SUVH5-like, 100% identity

```
g52579.t1       MEMGAAAAAKPSPREDGVRRHKVLVPWRFQPGYFRQPLKHAAPANGVAPNGDGGYTAAGV     60
g10978.t1       MEMGAAAAAKPSPREDGVRRHKVLVPWRFQPGYFRQPLKHAAPANGVAPNGDGGYTAAGV     60
                ************************************************************

g52579.t1       PDVNNCGSDGAPSPVGEANGSRDAKIFGSDGAPSPGKSGAVGGGEEQSDRCTRGQSLKSP     120
g10978.t1       PDVNNCGSDGAPSPVGEANGSRDAKIFGSDGAPSPGKSGAVGGGEEQSDRCTRGQSLKSP     120
                ************************************************************

g52579.t1       GVDNGGRPEPGNACNLGDSVRGGWVKSSPLEGTGNSRGGANDGGEAVAGDDCNMGSSNCD     180
g10978.t1       GVDNGGRPEPGNACNLGDSVRGGWVKSSPLEGTGNSRGGANDGGEAVAGDDCNMGSSNCD     180
                ************************************************************

g52579.t1       GSLKDVGTQDFTGAGDGAACDPEVIEITEECFAKGSKKPFVDQTGSKSNGSASGLRSEDP     240
g10978.t1       GSLKDVGTQDFTGAGDGAACDPEVIEITEECFAKGSKKPFVDQTGSKSNGSASGLRSEDP     240
                ************************************************************

g52579.t1       EGNVGLGDSAYHAAKGCSMGDEAAKENDATAKGCSSATPGSNGNGTYVRKGRKVGVPWRF     300
g10978.t1       EGNVGLGDSAYHAAKGCSMGDEAAKENDATAKGCSSATPGSNGNGTYVRKGRKVGVPWRF     300
                ************************************************************

g52579.t1       QVGYKRSFSDAFDSNNGSPDRPAYKFDGSSTQCTPTTRSSVRCYASSHSGVRVSAMHNLS     360
g10978.t1       QVGYKRSFSDAFDSNNGSPDRPAYKFDGSSTQCTPTTRSSVRCYASSHSGVRVSAMHNLS     360
                ************************************************************

g52579.t1       MKGENGTGTECKKRKTDSDYQDEVMPNNGDPIVRESIMRSLQDLRLIYREILDEEEDNSR     420
g10978.t1       MKGENGTGTECKKRKTDSDYQDEVMPNNGDPIVRESIMRSLQDLRLIYREILDEEEDNSR     420
                ************************************************************

g52579.t1       EKVINNGADMRAYKIFRERFSTEFDDEKYIGSVPGIYPGDIFHLRVELCVVGLHRQHRLG     480
g10978.t1       EKVINNGADMRAYKIFRERFSTEFDDEKYIGSVPGIYPGDIFHLRVELCVVGLHRQHRLG     480
                ************************************************************

g52579.t1       IDCTKKDDGITVAVSIVSCAQSSDAKYNLDVLVYTGPVAVTVNQRIEGTNLALKKSMDTS     540
g10978.t1       IDCTKKDDGITVAVSIVSCAQSSDAKYNLDVLVYTGPVAVTVNQRIEGTNLALKKSMDTS     540
                ************************************************************

g52579.t1       TPVRVIHGFTTKNGKKKFPIYIYGGLYLVEKYWREKEHGDRYVYMFRLRRMKGQKHIEIQ     600
g10978.t1       TPVRVIHGFTTKNGKKKFPIYIYGGLYLVEKYWREKEHGDRYVYMFRLRRMKGQKHIEIQ     600
                ************************************************************

g52579.t1       EILQTGDSGSNDSVIIKDLSLGLERVPVPVVNKISDECPMPYRYTSHLQYPRNYRPTPPA     660
g10978.t1       EILQTGDSGSNDSVIIKDLSLGLERVPVPVVNKISDECPMPYRYTSHLQYPRNYRPTPPA     660
                ************************************************************
```

```
g52579.t1      GCGCVDGCSDSKKCACAMKNGGEIPFNDKGRILEAKPLVYECGPSCKCPPTCHNRVGQHG      720
g10978.t1      GCGCVDGCSDSKKCACAMKNGGEIPFNDKGRILEAKPLVYECGPSCKCPPTCHNRVGQHG      720
               ************************************************************

g52579.t1      LKFRLQIFKTKSMGWGVRTLDFIPSGSFVCEYIGEVLEDEEAQKRTNDEYLFAIGHNYYD      780
g10978.t1      LKFRLQIFKTKSMGWGVRTLDFIPSGSFVCEYIGEVLEDEEAQKRTNDEYLFAIGHNYYD      780
               ************************************************************

g52579.t1      ESLWEGLSRSIPSLQKGPGKDDETGFAVDASEMGNFAKFINHSCTPNIYAQNVLYDHEDI      840
g10978.t1      ESLWEGLSRSIPSLQKGPGKDDETGFAVDASEMGNFAKFINHSCTPNIYAQNVLYDHEDI      840
               ************************************************************

g52579.t1      SVPHIMFFACDDIRPNQELLYHYNYKIDQVHDANGNIKKKKCLCGSVECDGWLY*   894
g10978.t1      SVPHIMFFACDDIRPNQELLYHYNYKIDQVHDANGNIKKKKCLCGSVECDGWLY*   894
               *******************************************************
```

tubulin beta-1 chain – 96.50% identity

```
g28522_B       MREILHIQGGQCGNQIGAKFWEVVCAEHGIDATGRYGGDSDLQLERVNVYYNEASCGRFV      60
g13706_A       MREILHIQGGQCGNQIGAKFWEVICDEHGIDHTGKYAGDSDLQLERINVYYNEASGGRYV      60
               ***********************:* ***** **:*.*********:******** **:*

g28522_B       PRAVLMDLEPGTMDSVRSGPYGHIFRPDNFVFGQSGAGNNWAKGHYTEGAELIDSVLDVV      120
g13706_A       PRAVLMDLEPGTMDSVRSGPYGQIFRPDNFVFGQSGAGNNWAKGHYTEGAELIDSVLDVV      120
               **********************:*************************************

g28522_B       RKEAENCDCLQGFQVCHSLGGGTGSGMGTLLISKIREEYPDRMMLTFSVFPSPKVSDTVV      180
g13706_A       RKEAENCDCLQGFQVCHSLGGGTGSGMGTLLISKIREEYPDRMMLTFSVFPSPKVSDTVV      180
               ************************************************************

g28522_B       EPYNATLSVHQLVENADECMVLDNEALYDICFRTLKLTTPSFGDLNHLISATMSGVTCCL      240
g13706_A       EPYNATLSVHQLVENADECMVLDNEALYDICFRTLKLATPTFGDLNHLISATMSGVTCCL      240
               *************************************:**:*******************

g28522_B       RFPGQLNSDLRKLAVNLIPFPRLHFFMVGFAPLTSRGSQQYRALTVPELTQQMWDAKNMM      300
g13706_A       RFPGQLNSDLRKLAVNLIPFPRLHFFMVGFAPLTSRGSQQYRALTVPELTQQMWDAKNMM      300
               ************************************************************

g28522_B       CAADPRHGRYLTASAMFRGKMSTKEVDEQMLNVQNKNSSYFVEWIPNNVKSTVCDIPPTG      360
g13706_A       CAADPRHGRYLTASAMFRGKMSTKEVDEQMLNVQNKNSSYFVEWIPNNVKSSVCDIPPTG      360
               ***************************************************:********

g28522_B       LKMASTFIGNSTSIQEMFRRVSEQFTAMFRRKAFLHWYTGEGMDEMEFTEAESNMKDLVF      420
g13706_A       LKMASTFIGNSTSIQEMFRRVSEQFTAMFRRKAFLHWYTGEGMDEMEFTEAESNMNDLVA      420
               *******************************************************:***

g28522_B       EYQVYQDAIVDEVGEYEDEEEADLQD*      446
g13706_A       EYQQYQDATADDEEEYEDEEEEEEAA*-     445
               *** **** .*:  ******* :
```

tubulin alpha-3 chain – 97.98% identity

```
g24105_A       MRECISVHIGQAGIQVGNACWELYCLEHGIQPDGQVPGDKTAGHHDDAFSTFFSQTGAGK      60
g8510_B        MRECISVHIGQAGIQVGNACWELYCLEHGIQPDGQVPGDETAGHHDDAFSTFFSQTGAGK      60
               ***************************************:********************

g24105_A       HVPRAIFVDLEPTVIDEVRTGTYRQLFHPEQLISGKEDAANNFARGHYTIGKEIVDLCLD      120
g8510_B        HVPRAIFVDLEPTVIDEVRTGTYRQLFHPEQLISGKEDAANNFARGHYTIGKEIVDLCLD      120
               ************************************************************

g24105_A       RIRKLADNCTGLQGFLVFNAVGGGTGSGLGSLLLERLSVEYGKKSKLGFTVYPSPQVSTS      180
g8510_B        HIRKLADNCTGLQGFLVFNAVGGGTGSGLGSLLLERLSVEYGKKSKLGFTVYPSPQVSTS      180
               :***********************************************************

g24105_A       VVEPYNSVLSTHSLLEHTDVSILLDNEAIYDICRRSLDIERPNYSNLNRLVSQVISSLTA      240
```

```
g8510_B         VVEPYNSVLSTHYLLEHTDVSILLDNEAIYDICRRSLDIERPNYSNLNRLVSQVISSLTT      240
                ************ **********************************************:

g24105_A        SLRFDGALNVDVNEFQTNLVPYPRIHFMLSSYAPVISSAKAFHEQLSVAEITSSAFEPAS      300
g8510_B         SLRFDGAINVDVNEFQTNLVPYPRIHFMLSSYAPVISSAKAFHEQLSVAEITSSAFEPAS      300
                *******:****************************************************

g24105_A        MMVKCDPRHGKYMACCLMYRGDVVPKDVNAAVSLIKTKRTIQFVDWCPTGFKCGINYQAP      360
g8510_B         MMVKCDPRQGKYMACCLMYRGDVVPKDVNAAVGLTKTKRTIQFVDWCPTGFKCGINYQAP      360
                ********:***********************.* *************************

g24105_A        TVVPGADLAKVQRAVCMISNSTSVVEVFSRINSKFDLMYAKRAFVHWYVGEGMEEGEFSE      420
g8510_B         TVVPGADLAKVQRAVCMISNSTSVVEVFSRINSKFDLMYAKRAFVHWYVGEGMEEGEFSD      420
                ***********************************************************:

g24105_A        AREDLAALEKDYEEVAAEGGGDEGEEDEEY*-----------------------------      450
g8510_B         AREDLAALEKDYEEVAAEGGGDEGEVVQRRRDALQCGDGLTVGINMVARSLRAGPGGYVL      480
                *************************  :.

g24105_A        ----------------   450
g8510_B         VVVGGRYVSGAAAAW*   495
```

mRNA turnover protein 4 homolog – 83.78% identity

```
g35119_B        ------------------------------------------------------------      0
g16140_A        MPKSKRNRPVTLSKTKKKPGLERKGKVVAEIKDAIDRYSSAYVFTYDNMRNQKLKDLREQ      60

g35119_B        ------------------------------------------------------------      0
g16140_A        LKSSSRIFLAGKKVMQIALGRSPADEAKTGLHKLSKFLQGDSGLFFTNLPRDDVERMFRE      120

g35119_B        -----MKR---RSSKIVPMRWGERQIF-------VDFFCNPERLKNQVRELTSRVKALQN      45
g16140_A        FEEHDFARTGSTATETVELKEGPLEQFTHEMEPFLRKQGLPVRLNKGV------------      168
                     : *    ::: * :: *  : *       :     * **:: *

g35119_B        CSCTLVLHVIHFLCIKFNFTPTSSWGRCAGTRLLGVQMVTLRLNLVCCWSCDDFKVYKEG      105
g16140_A        ----IELVADHVVCEEG---KPLSPEAAQTLRLLGIQMATFRLYLVCRWSCDDFEVYKEG      221
                    : * . *.:* :       *   .   ****:**.*:** *** ******:*****

g35119_B        LMHLGADDFLLSLCFLYTRQHFAVLTRTVFGSLCVAM* 142
g16140_A        LMHLGADDSS*--------------------------- 231
                ********
```

ATP-dependent RNA helicase DEAH11 – 98.91% identity

```
g1204_B         MRRSQDRGLLRPPDWVPRPPP--HHRDHHYHNEHRYPPHSHPHRDRHYSAERRYQPRAQQ      58
g46421_A        MRRSQDRGLLRPPDWVPRPPPQHHHRDHHYHNEHRHPPHSHPHRDRHYSAERRYQPRAQQ      60
                *********************  ************:************************

g1204_B         PSPPPSQFEVLLVRPGPDLSAPTAIEVEGLVAGLPSPPPASVSVHSSGRHAARLVFASVS      118
g46421_A        PSPPPSQFEVLLVRPGPDLSAPTAIEVEALVAGLPPPAPASVSVHSSGRHAARLAFASVS      120
                ****************************.****** * ****************.*****

g1204_B         DAAAAARQLWALRLEGLHLLALDLPHAAVAAHAKPLIASLFSDHASRLLDSDLVAVSAAR      178
g46421_A        AAAAAARQLWALRLEGLHLLALDLPHAAVAAHAKPLIASLFADHASRLLDSDLVAVSAAR      180
                 ****************************************:******************

g1204_B         SADLAASIRDVKRRLAGRNRVRDFHQLDLEKKTLESEKELVDAKIAEYKEAMLSIQRAML      238
g46421_A        SADLAASIRDAKRRLGGRNRVRDFHQLDLEKKTLESEKELVDAKIAEYKEAMLSIQRAML      240
                **********.****.********************************************

g1204_B         RRSGDKEEGVHLFGAVEGADVDFVRVHMMLLRECRRLKEGLPIYAYRRKILNHILANQAM      298
g46421_A        RGSGDKEEGVHLFGAVEGADVDFVRVHMMLLRECRRLKEGLPIYAYRRRILNHILANQAM      300
```

```
              * ********************************************** :***********

g1204_B       VLIGETGSGKSTQLVQFLADSGLAGGRSIVCTQPRKLAAISLAHRVDEESKGCYGDSSVM     358
g46421_A      VLIGETGSGKSTQLVQFLADSGLAGGRSIVCTQPRKLAAISLAHRVDEESKGCYGDSSVM     360
              ************************************************************

g1204_B       SYSTLLNSQGFGTKIIFTTDSCLLHNCMSDMSLDGISYVIIDEAHERSLNTDLLLAMIKK     418
g46421_A      SYSTLLNSQGFGTKIIFTTDSCLLHNCMSDMSLDGISYVIIDEAHERSLNTDLLLAMIKK     420
              ************************************************************

g1204_B       KLLDRLDLRLIIMSATADADRLAEYFFGCQTFHVKGRTFPVEIKYVPDISAEASLNSIPS     478
g46421_A      KLLDRLDLRLIIMSATADADRLAEYFFGCQTFHVKGRTFPVEIKYVPDISAEASLNSVPS     480
              *********************************************************:**

g1204_B       MSSVASAAPSYVTDVVQMVNIIHKNEEEGAILAFLTSQLEVEWACETFSDPNAVVLPMHG     538
g46421_A      MSSVASAAPSYVTDVVQMVNIIHKNEEEGAILAFLTSQLEVEWACETFSDPNAVVLPMHG     540
              ************************************************************

g1204_B       KLSSIEQNLVFQSYPGKRKIIFCTNIAETSLTIKEVKYVVDCGLAKEYRFVPSSGLNVLK     598
g46421_A      KLSSIEQNLVFQSYPGKRKIIFCTNIAETSLTIKEVKYVVDCGLAKEYRFVPSSGLNVLK     600
              ************************************************************

g1204_B       VNWISQSSANQRAGRAGRTGAGKCYRLYPESDFGLMEAHQEPEIRKVHLGTAVLRILALG     658
g46421_A      VNWISQSSANQRAGRAGRTGAGKCYRLYPESDFGLMEAHQEPEIRKVHLGTAVLRILALG     660
              ************************************************************

g1204_B       VPDVKYFEFVDAPDPEAINMAVHNLEQLGAIKYKCSGFELTDTGRDLVKLGIEPRLGKIM     718
g46421_A      VPDVKYFEFIDAPDPEAINMAVHNLEQLGAIKYKCSGFELTDTGRDLVKLGIEPRLGKIM     720
              *********:**************************************************

g1204_B       LDCFSYGLMKEGLVLASVMANASSIFCRVGTNEEKYKADRLKVPFCHPDGDLFTSLAVYK     778
g46421_A      LDCFSYGLMKEGLVLASVMANASSIFCRVGTNEEKYKADRLKVPFCHPDGDLFTSLAVYK     780
              ************************************************************

g1204_B       KWEAGPDNKNMWCWQNSINAKTLRRCQETISELEKCLKHELNTIVPSYWSWNPEKPTMHD     838
g46421_A      KWEAGPDNKNMWCWQNSINAKTLRRCQETISELEKCLKHELNTIVPSYWSWNPEKPTMHD     840
              ************************************************************

g1204_B       TTLKKIILSSLRGNLAMFSGHEKFGYQVISADQPVQLHPSCSLLTYGSKPEWVVFSEILS     898
g46421_A      TTLKKIILSSLRGNLAMFSGHEKFGYQVISADQPVQLHPSCSLLTYGSKPEWVVFSEILS     900
              ************************************************************

g1204_B       VPNQYLVCVTAVDRNEVCTVNSMSFIEQVEESKLQRKVITGIGNKSLRRFCGKSGQNLQK     958
g46421_A      VPNQYLVCVTAVDRNEVCTVNSMSFIEQVEESKLQRKVITGIGNKSLRRFCGKSGQNLQK     960
              ************************************************************

g1204_B       IVSLLREDCRDDRIMVDLDFSSSEVLLFAKEHDMETVFCVVNDALELEAKMLSDECDERR     1018
g46421_A      IVSLLREDCRDDRIMVDLDFSSSEVLLFAKEHDMETVFCVVNDALELEAKMLSDECDERR     1020
              ************************************************************

g1204_B       PGGSTIALFGSGAEIKHLELGKRCLTVEIMHQNARDIDEKELIGLVYSHVPGIANFHKLG     1078
g46421_A      PGGSTIALFGSGAEIKHLELGKRCLTVEILHQNARDIDEKELIGLVYSHVPGIANFHKLG     1080
              *****************************:******************************

g1204_B       NFQSNTDETKWGRFTFLKPDYADDAISKLNGIEFHGSSLKVGHVSTYNHPGLPFPAVRAK     1138
g46421_A      NFQSNTDETKWGRFTFLKPDYADDAISKLNGIEFHGSSLKVGHVSTYNHPGLPFPAVRAK     1140
              ************************************************************

g1204_B       VSWPRKPSRGLALVTCASGEAEFIVKDCFALGVGGRYINCEVSKKFANCVFVRGVPLHVT     1198
g46421_A      VSWPRKPSRGLALVTCASGEAEFIVKDCFALGVGGRYINCEVSKKFANCVFVRGVPLHVT     1200
              ************************************************************

g1204_B       EPELYDAFRSTTTRRILDIRLLRGAPTASSSDSECAEALMRAISLFMPNRNFPGQNFRVH     1258
g46421_A      EPELYDAFRSTTTRRILDIRLLRGAPTASSSDSECAEALMRAISLFMPNRNFPGQNFRVH     1260
              ************************************************************

g1204_B       VIPPEEKDSMMRATITFDGSFHREAARALDHLQGSVLPCCLPWQIIQCQHVFHSTVSCPM     1318
g46421_A      VIPPEEKDSMMRATITFDGSFHREAARALDHLQGSVLPCCLPWQIIQCQHVFHSTVSCPM     1320
```

```
                ************************************************************

g1204_B         RIYNVISQEVGVLLESFRSEKGVSYNLEKNENGHFRVKLTANATKTIADLRRPLELLMKG      1378
g46421_A        RIYNVISQEVGVLLESFRSEKGVSYNLEKNENGHFRVKLTANATKTIADLRRPLELLMKG      1380
                ************************************************************

g1204_B         KIINHPDLMLSTVQLLWSRDGMEHLKSVEQETGTYILYDRQSRNIKVFGSTDKVAAAEEK      1438
g46421_A        KIINHPDLMLSTVQLLWSRDGMEHLKSVEQETGTYILYDRQSRNIKVFGSTDKVAAAEEK      1440
                ************************************************************

g1204_B         LVRALVQLHEKKPLEVCLRGRNLPPNLMKEVIKKFGADLEGLKTEVPAVDLQLNTRKQTL      1498
g46421_A        LVRALVQLHEKKPLEVCLRGRNLPPNLMKEVIKKFGADLEGLKSEVPAVDLQLNTRKQTL      1500
                *******************************************:****************

g1204_B         YVRGSKEDKQRVEEMISELIASSDHNAPLPSKNACPICLCELEDPFKLESCGHMFCFACL      1558
g46421_A        YVRGSKEDKQRVEEMISELIASSDHNAPLPSKNACPICLCELEDPFKLESCGHMFCFACL      1560
                ************************************************************

g1204_B         VDQCESAMKSQGGFPLCCLKNGCKNLLLLADLRSLVPDKLDELFRASLNAFVASSAGVYR      1618
g46421_A        VDQCESAMKSQDGFPLCCLKNGCKNLLLLADLRSLVPDKLDELFRASLNAFVASSAGLYR      1620
                ***********.*********************************************:**

g1204_B         FCPTPDCTSIYQVGAAGAEDKPFVCGACSVETCTKCHLEYHPFISCEAYKEYKADPTDAT      1678
g46421_A        FCPTPDCTSIYQVGAAGAEDKPFVCGACSVETCTKCHLEYHPFISCEAYKEYKADPTDAT      1680
                ************************************************************

g1204_B         LLEWRKGKENVKNCPSCGYTIEKAEGCNHVECRCGSHICWNCLESFKSSEECYGHLRSVH      1738
g46421_A        LLEWRKGKENVKNCPSCGYTIEKAEGCNHVECRCGSHICWNCLESFKSSEECYGHLRSVH      1740
                ************************************************************

g1204_B         LAYV* 1742
g46421_A        LAYV* 1744
                *****
```

CENH3 *Aegilops speltoides*

```
Aeg_CENH3_A_g18195      MARTKHPAVRKTKA-----PPKKQLGPRPAQRRQETDGAGTSATPRRAGRAAAPG--AAE   53
Aeg_CENH3_B_g94071      MTRTKKPPVSKLKMTRTKQPPVSKLKVRA------A-----------DGSARSPGGTQQT   43
                        *:***:* * * *      ** .:*  *       :            * * :**

Aeg_CENH3_A_g18195      GATGQPKQRKPHRFRPGTVALREIRKYQKSVDFLIPFAPFVRLIKEVTDFFCPEISRWTP   113
Aeg_CENH3_B_g94071      AASGQPRQRKPHRFRPGTVAQREIRKYQKSVDLLIPLAPFVRLIKEITNDFREG-IRFTP   102
                        .*:***:************* ***********:***:*********:*: *     *:**

Aeg_CENH3_A_g18195      QALVAIQEAAEYHLVDVFERANHCAIHAKRVTVMQKDIQLARRIGGRRLW*     163
Aeg_CENH3_B_g94071      GALMTIQEAAEYHLVDEFERANHCANHAKRVTVTLKDIELARLIGGRRLW*     152
                         **::*********** ******** *******  ***:*** ********
```

CENH3 *Zea mays*

```
Zea_mays_CenH3_A      MARTKHQAVRKTAEKPKKKLQFERSGGASTSATPERAAGTGGRAASGGDSVKKTKPRHRW     60
Zea_mays_CenH3_B      MARTKHQAVRKPAEKPKKKLQFERS-------------------------VKKTKPRHRW     35
                      *********** *************                         **********

Zea_mays_CenH3_A      RPGTVALREIRKYQKSTEPLIPFAPFVRVVRELTNFVTNGKVERYTAEALLALQEAAEFH     120
Zea_mays_CenH3_B      RPGTVALREIRKYQKSTEPLIPFAPFVRVVRELTNFLTNGKVERYTPEALLALQEAAEFH     95
                      ************************************:********* *************

Zea_mays_CenH3_A      LIELFEMANLCAIHAKRVTIMQKDIQLARRIGGRRWA 157
Zea_mays_CenH3_B      LIELFEIANLCAIHAKRVTVMHKDIQLARRIGGRRWA 132
                      ******:************:*:***************
```
