# Supplementary material for: Sorghum embryos undergoing B chromosome elimination express B-variants of mitotic-related genes
Source: Genome Biol. 2025 Dec 24;27:8. doi: 10.1186/s13059-025-03915-w (PMC12849586; doi:10.1186/s13059-025-03915-w)
Supplement: Supplementary file 5 — Additional file 5. Supplementary Document (file contains full figure of phylogenic tree of CENH3 proteins). [file 13059_2025_3915_MOESM5_ESM.pdf]

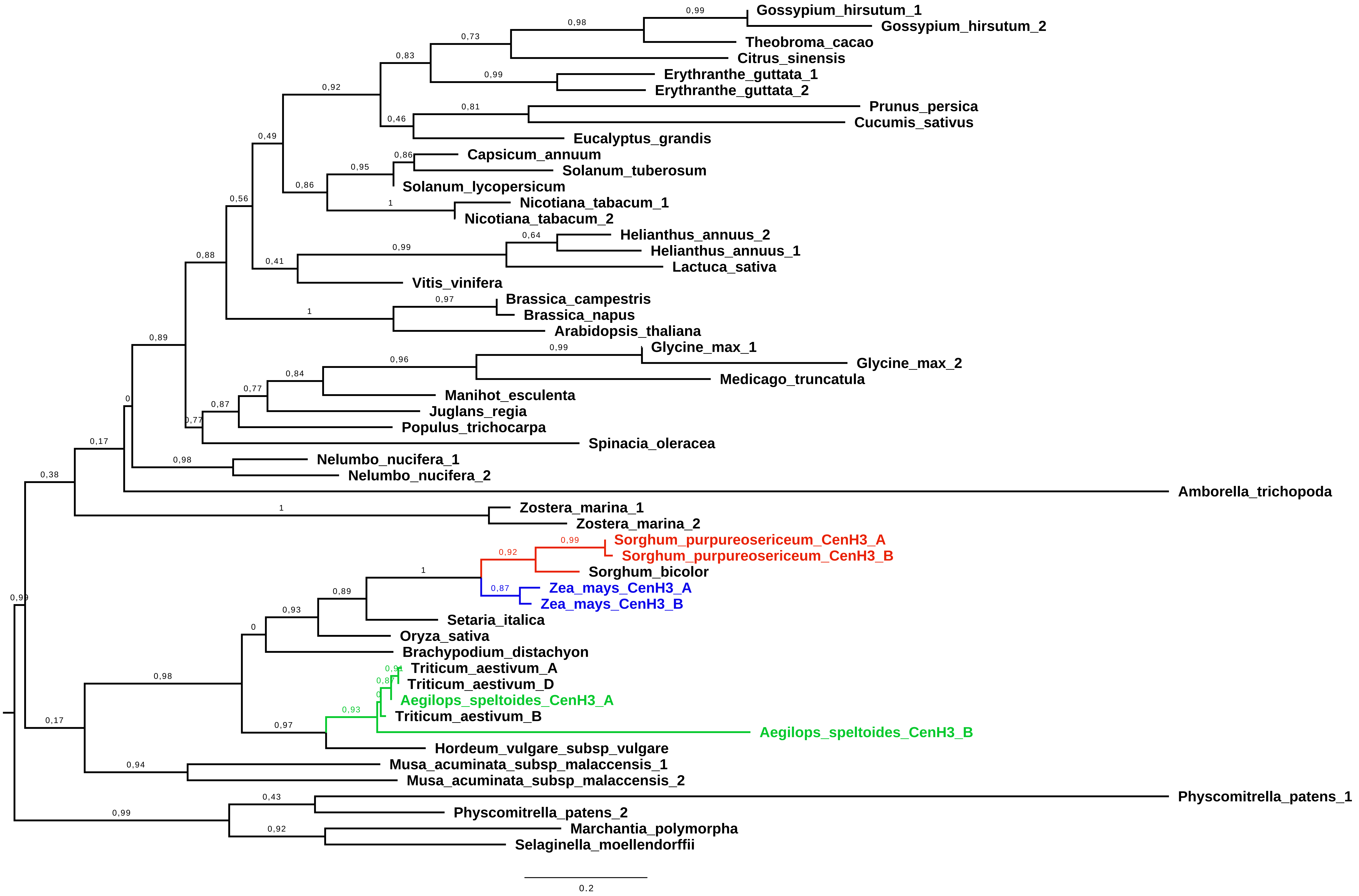

Additional file 5: Complete phylogenetic tree of CENH3 Proteins. Proteins from species with B chromosomes are highlighted in red (Sorghum purpureosericeum), green (Aegilops speltoides), and blue (Zea mays). Only protein sequences belonging to the PANTHER subfamilies PTHR11426:SF223 and PTHR11426:SF277 were included in the analysis.
